# Monitoring Substance Use with Fitbit Biosignals: A Case Study on Training Deep Learning Models Using Ecological Momentary Assessments and Passive Sensing

**DOI:** 10.3390/ai5040131

**Published:** 2024-12-03

**Authors:** Shizhe Li, Chunzhi Fan, Ali Kargarandehkordi, Yinan Sun, Christopher Slade, Aditi Jaiswal, Roberto M. Benzo, Kristina T. Phillips, Peter Washington

**Affiliations:** 1 Department of Statistics, Stanford University, Stanford, CA 94305, USA; 2 Institute for Computational and Mathematical Engineering, Stanford University, Stanford, CA 94305, USA; 3 Department of Information and Computer Sciences, University of Hawaii at Manoa, Honolulu, HI 96822, USA; 4 Communication & Information Sciences, University of Hawaii at Manoa, Honolulu, HI 96822, USA; 5 Division of Cancer Prevention and Control, The Ohio State University College of Medicine, Columbus, OH 43210, USA; 6 Center for Integrated Health Care Research, Kaiser Permanente Hawaii, Honolulu, HI 96817, USA; 7 Department of Health Systems Science, Kaiser Permanente Bernard J. Tyson School of Medicine, Pasadena, CA 91101, USA; 8 Division of Clinical Informatics and Digital Transformation, Department of Medicine, University of California, San Francisco, CA 94143, USA

**Keywords:** wearables, Fitbit, substance use, self-supervised learning, personalized models, remote monitoring

## Abstract

Substance use disorders affect 17.3% of Americans. Digital health solutions that use machine learning to detect substance use from wearable biosignal data can eventually pave the way for real-time digital interventions. However, difficulties in addressing severe between-subject data heterogeneity have hampered the adaptation of machine learning approaches for substance use detection, necessitating more robust technological solutions. We tested the utility of personalized machine learning using participant-specific convolutional neural networks (CNNs) enhanced with self-supervised learning (SSL) to detect drug use. In a pilot feasibility study, we collected data from 9 participants using Fitbit Charge 5 devices, supplemented by ecological momentary assessments to collect real-time labels of substance use. We implemented a baseline 1D-CNN model with traditional supervised learning and an experimental SSL-enhanced model to improve individualized feature extraction under limited label conditions. Results: Among the 9 participants, we achieved an average area under the receiver operating characteristic curve score across participants of 0.695 for the supervised CNNs and 0.729 for the SSL models. Strategic selection of an optimal threshold enabled us to optimize either sensitivity or specificity while maintaining reasonable performance for the other metric. Conclusion: These findings suggest that Fitbit data have the potential to enhance substance use monitoring systems. However, the small sample size in this study limits its generalizability to diverse populations, so we call for future research that explores SSL-powered personalization at a larger scale.

## 1. Introduction

Substance use is a major public health issue in the United States. According to the National Survey on Drug Use and Health [1], in 2022, approximately 48.7 million Americans aged 12 or older, or 17.3 percent, had a reported substance use disorder. Given the extensive burden of substance abuse on personal and public health, there is considerable interest in innovative solutions to mitigate its effects. Wearable technologies such as smartwatches have emerged as a promising monitoring tool, with about one-third of U.S. adults using these devices for health monitoring [2]. These relatively low-cost, non-invasive devices hold potential for sensing real-time use and cravings, potentially leading to digital interventions that can reduce heavy use, avoid relapse, and monitor for potential overdoses. By providing insights through continuous physiological monitoring, wearables have the potential to enhance treatment strategies and improve outcomes for individuals with substance use disorders [3].

There has been a surge in research interest around leveraging artificial intelligence (AI) to develop models using biosensor data for the detection of substance use [4]. Wrist-worn or mobile devices equipped with biosensors usually collect data that continuously monitor physiological and behavioral metrics. These data can include HR, movement patterns, skin temperature, and electrodermal activity, providing insights into an individual’s physical and behavioral state [5]. Remote ecological momentary assessments (EMAs), or in-the-wild self-reports via means such as a smartphone, can gather prediction labels while participants carry out their everyday activities, offering contextually relevant data [6–8]. EMAs have been used to study substance use, as it is known to be triggered by episodic behavior and contextual factors [9–12]. By capturing dynamic, real-time interactions between behavior and physiological factors, EMAs can offer valuable insights into substance use. While many computational techniques can efficiently process and categorize data nearly in real-time, they often fall short in terms of adjusting to the heterogeneity of biosignal data both within and between subjects, indicating a need for more adaptable AI solutions [4]. To address this issue, we propose personalized AI models that enable the models to adapt dynamically to each patient’s unique characteristics.

In this case study, we aimed to develop personalized machine learning models for drug use prediction, as measured by EMAs, from Fitbit biosignal data. We gathered data from 9 adults with varied drug use patterns and health conditions, all recruited from two local programs in Hawaii, including a harm reduction outreach program and a substance use treatment facility, as well as students and staff from the University of Hawaii community. The participants continuously tracked their physiological metrics using commercially available, low-cost Fitbit Charge 5 devices. Participants also logged specific instances of substance use through EMAs administered via a mobile app. We trained two convolutional neural networks (CNNs) for each participant: (1) a control model using traditional supervised learning and (2) an experimental model optimized to enhance personalized feature extraction via self-supervised learning (SSL) applied at the level of individual biosignal data.

We contribute the following: (1) We demonstrate that commercially available and inexpensive non-research devices with few sensors, like the Fitbit Charge 5, can still capture significant predictive signals related to substance use. (2) We develop and validate personalized models that are tailored to the unique physiological and behavioral patterns of each participant. (3) We explore whether SSL can boost the predictive capabilities of our models. (4) We conduct a comprehensive analysis of the trade-offs between sensitivity and specificity at various thresholds.

## 2. Related Work

### 2.1. Wearable Biosensors for Substance Detection

Our work builds upon a growing field of research on the use of wearable biosignals for substance use prediction. Researchers have applied wearable biosensors to substance use detection in a variety of settings, from emergency departments to natural environments, providing objective data [13,14]. Carreiro et al. demonstrated how these devices can differentiate between stress and craving signals [15]. Various machine learning techniques, such as support vector machines (SVMs) and neural networks, have enhanced the automated detection of substance use behaviors through smartphone sensors and other biosensors [16,17]. For example, Natarajan et al. applied logistic regression to detect cocaine use with wearable electrocardiogram sensors [18], while Mahmud et al. employed gradient boosted trees [19] and Gullapalli et al. applied temporal convolutional attention networks [20] for broad drug use detection. Studies have also employed random forests for detecting kratom intoxication [21] and SVM-based methods for monitoring non-adherence in opioid surveillance [22]. Wang et al. developed a method that utilizes data streams from wearable biosensors monitoring sympathetic nervous system activity, showcasing the potential of biosensors in real-time drug detection [23].

### 2.2. SSL and Personalization

Despite recent advancements in wearable biosensors and machine learning, substantial challenges remain for achieving clinical-grade performance. Current algorithms and models frequently struggle to adapt to new conditions and manage dynamic changes in sensor data. This has catalyzed the development of more adaptable AI solutions, such as SSL and personalized modeling, which can significantly enhance the adaptability and effectiveness of monitoring technologies.

Personalized deep learning models have effectively predicted changes in anxiety levels, demonstrating the practicality and impact of using personally tailored models for mental health monitoring [7]. Combining personalization with SSL predicts stress events on public datasets more accurately than baseline approaches, showcasing the benefits of SSL in enhancing personalized learning models [5,24,25].

Research into personalized models using SSL have further explored the potential of deep learning techniques to predict complex physiological events such as stress-induced blood pressure spikes [26]. Additionally, researchers have used personalized models to detect collaborative non-adherence in opioid surveillance [22]. These developments offer promising future avenues for personalized healthcare interventions.

## 3. Materials and Methods

**Figure 1.**
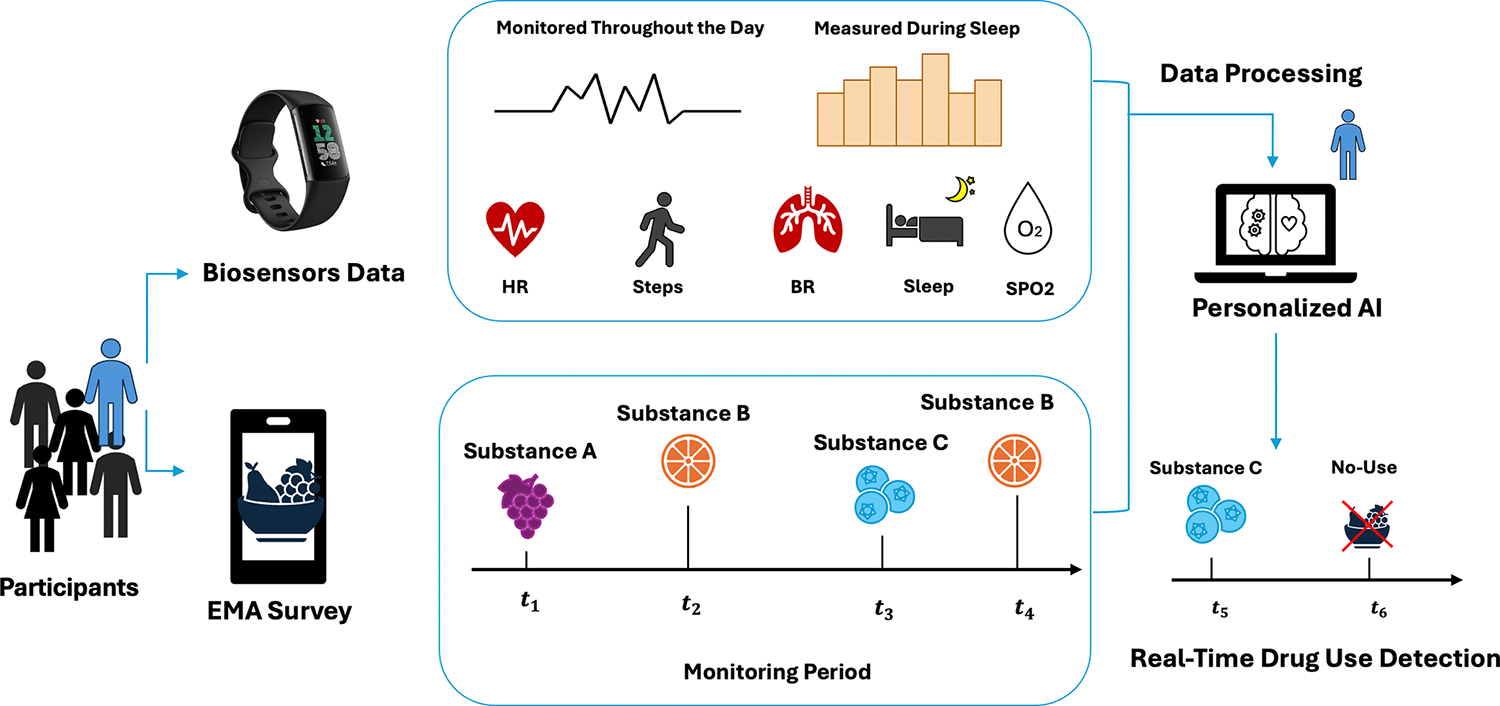
Study overview. We recruited participants and equipped them with Fitbits collecting various biosensor data, including HR, steps taken, BR, sleep patterns, and SpO_2_. Concurrently, participants completed EMAs via a custom mobile app, recording each substance use event over the monitoring period. We then analyzed these data using personalized deep learning models to detect substance use based on biosensor data from the Fitbit. To protect patient privacy and to avoid asking participants to self-report illegal activity, we gave participants the option to record fruit code names rather than substance names, and the participants eligible for our analysis chose this option.

This pilot feasibility study aimed to utilize wearable Fitbit sensors combined with machine learning to predict real-time drug use (Figure 1). We collected data from 9 participants recruited through two local programs in Hawaii: a harm reduction outreach initiative and a substance use treatment facility. We also recruited broadly at the University of Hawaii, including both students and staff. We equipped all participants with Fitbit devices that continuously monitored heart rate (HR) and step count during waking hours as well as breathing rate (BR), steps, sleep quality, and blood oxygen saturation (SpO_2_) during sleep. Alongside biosensor data, we asked the participants to complete EMA surveys 4 times a day to provide labels of substance use and cravings. We trained personalized AI models to predict real-time drug use from a 1 h time window of Fitbit data.

9 participants provided data specific to drug use patterns and recorded at least ten instances of drug use during the monitoring period, thus making them eligible for inclusion in our analyses. These individuals were actively using at least one of the following substances during the study period: methamphetamine (meth), alcohol, cannabis, opioids, cocaine, sedatives/benzodiazepines, nicotine, mushrooms, or Gamma-hydroxybutyrate (GHB) at least twice per week. The 9 participants consisted of 3 men, 5 women, and 1 non-binary individual, with ages ranging from 23 to 55 years and averaging 36.1 years. The race and ethnicity breakdown of the participants was as follows: 2 white, 2 native Hawaiian, 1 Japanese, 1 Hispanic (Mexican), 1 Samoan or Tongan, and 1 Filipino. All were residents of Oahu, Hawaii and had consistent access to a smartphone with data connectivity throughout the 4-week study period.

During the monitoring period, participants wore a Fitbit Charge 5 watch continuously, removing it only for necessary activities such as exposure to water (e.g., showering, swimming, or surfing). We instructed them to wear the device 24 h a day to capture both daytime and nighttime physiological data, which included HR variability (HRV), BR, SpO_2_, and processed sleep quality data, collected primarily during nighttime rest. We captured and managed data via a bespoke app called BanAware, downloaded on participants’ personal smartphones. Developed for both iOS and Android platforms, the app facilitated the real-time monitoring and the collection of EMA data. Participants periodically recorded their substance use and related behaviors through the app while maintaining their normal routines to ensure the accurate capture of natural behavior patterns. Additionally, we required them to complete EMAs four times daily. We securely stored the data on Amazon Web Services (AWS), adhering to the Health Insurance Portability and Accountability Act (HIPAA) guidelines for encryption and security. We processed the data on an Elastic Cloud Compute (EC2) instance and used DynamoDB for storage, featuring mandatory server-side encryption to protect participant confidentiality. For detailed protocol specifications, see Sun et al. [27].

### 3.1. Biometric Features

We hypothesized that the biosignal data we collected would be useful for detecting substance use based on prior work [15,18,19,21,28]. During waking hours, the sensors measured HR and step count. We monitored BR, HRV, and SpO_2_ during sleep. Detailed BR analyses covered various sleep stages—deep sleep, REM (rapid eye movement), and light sleep—as well as cumulative metrics for entire sleep periods. Sleep metrics such as sleep efficiency, total sleep duration, minutes awake, and sleep onset time provided comprehensive insights into participants’ sleep patterns. We recorded SpO_2_ at one-minute intervals to monitor respiratory and cardiovascular health during the sleep period, and HRV features included the root mean square of successive differences (RMSSDs), low frequency (LF), high frequency (HF), and overall metric coverage.

Increases in HR are associated with drug craving [29]. Therefore, we recorded HR data throughout the day at one-minute intervals and then aggregated them into one-hour time steps to reduce noise. During these intervals, we calculated statistical metrics such as the mean, standard deviation, maximum, and dominant frequency. The dominant frequency within each time step was determined using the fast Fourier transform (FFT), mathematically represented as:

$$FFT(x)=\sum_{n=0}^{N-1}x(n)\cdot e^{-\frac{i2\pi kn}{N}},$$

This transformation converted the time-series HR data into the frequency domain, facilitating the examination of periodic patterns and rhythms across the hourly intervals. FFT allowed for the detection of recurring patterns that may not be immediately visible in the time domain. Frequency components provided deeper insights into anomalies or specific rhythms that may correspond to physiological changes associated with drug use.

Physical activity positively reduces drug cravings in individuals with substance use disorders by enhancing internal inhibition and resistance to drugs, with moderate- to high-intensity exercise proving most effective in supporting sports rehabilitation and strengthening the ability to inhibit cravings and manage drug-seeking behaviors [30,31]. For step data, we recorded measurements at one-minute intervals. In processing these data, we calculated the average and maximum step counts within each hourly interval. Additionally, we computed the total number of active minutes per hour, defined as minutes where the step count exceeded zero. This approach allowed for a detailed analysis of physical activity patterns throughout the day.

For the SpO_2_ data specifically collected during sleep periods, we used a binary marker to identify instances when SpO_2_ levels fell below 90%, indicating potential hypoxemia. The daily aggregations of these measurements included metrics such as the average SpO_2_, the dominant frequency of SpO_2_ values during the sleep period, and the total number of minutes per day with SpO_2_ levels below 90%. These metrics are commonly used for analyzing SpO_2_ [32].

Substance use, particularly involving alcohol, cannabis, cocaine, and opioids, can significantly disrupt sleep architecture [33,34]. Such substances can affect total sleep time, REM sleep, and sleep efficiency, leading to issues like increased sleep latency, reduced slow-wave sleep (SWS), and sleep fragmentation during withdrawal periods [35]. Stimulants like nicotine tend to increase HR and reduce HRV by activating the sympathetic nervous system, resulting in lower HRV [36,37]. Low HF-HRV is associated with more severe symptoms in individuals with substance use disorder than in those with PTSD [38]. We mapped these features to the previous day’s data and averaged them to correlate sleep-related data with the previous day’s drug use for predictive analysis. We measured BR and sleep-related metrics during each sleep cycle, while we recorded HRV every minute. We calculated the ratio of low to high frequency (L/H Ratio).

For each participant, we represented each of the features as a 1D time series for subsequent feature selection.

### 3.2. EMA Labels

The labeled data consisted of both “use” and “non-use” labels, generated based on drug use instances reported in EMA surveys. The “use” labels indicated the start time and type of drug used. Throughout the monitoring period, participants periodically completed EMA surveys while maintaining their daily routines to ensure the accurate recording of all drug use instances. In each EMA, the participants reported the approximate times (including the date, hour, and minute) of their drug use within the past 24 h via the smartphone app interface. For each reported drug use instance, we extended the corresponding label using a sliding window approach, aligning it with the average effect duration of the specific substance used. We aggregated each instance into a 1 h interval for noise reduction. We automatically labeled time periods without drug use as “non-use”.

### 3.3. Data Processing

We divided the time series data into overlapping windows of 12 time steps, with each step representing 1 h. We chose a temporal input length of 12 h for modeling short-term dependencies and capturing intraday patterns. As indicated by the data quality table, the drug use labels were limited relative to the large number of input features. To address this imbalance, we oversampled the “use” labels by increasing their frequency within local sections of the dataset while maintaining the temporal integrity of the time-series data.

**Figure 2.**
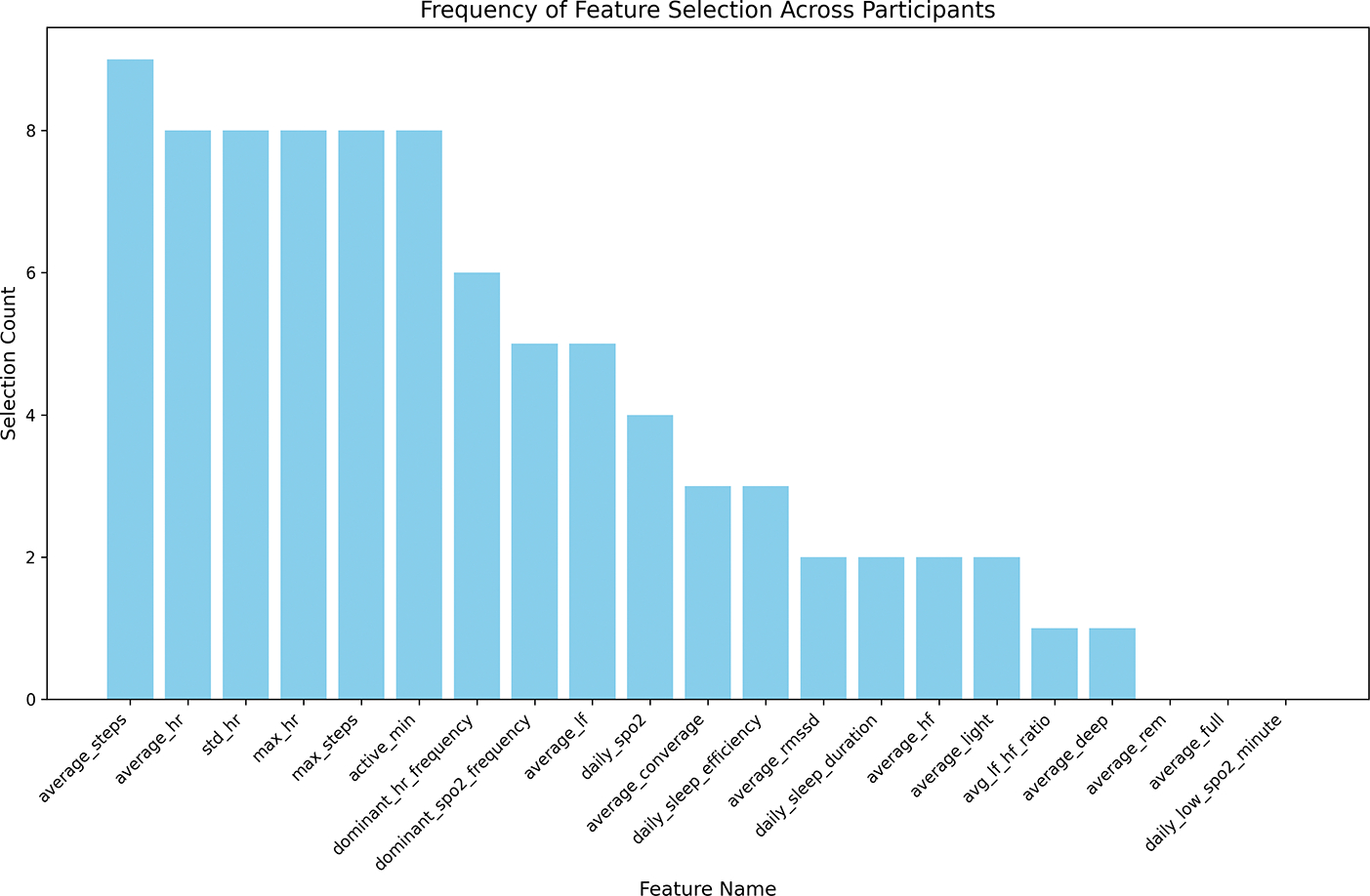
Distribution of each feature’s utilization. Features are ranked by their selection count using Gini impurity.

For feature selection, we used a random forest model to assess the feature importance based on reductions in Gini impurity. Given the variability among participants, we retained features exceeding the median importance score individually for each person, allowing the personalized refinement of the model to emphasize predictors critical for predictive accuracy in specific contexts. Figure 2 shows the distribution of feature utilization. Additionally, we imputed missing values in the dataset using a random forest model. Finally, we normalized the input features before training.

### 3.4. Model Architecture and Training

To identify patterns indicative of drug use, we developed two models using 1D convolutional neural networks (1D-CNN) for each participant: a control model utilizing traditional supervised learning and an experimental model employing SSL to enhance feature extraction and pattern recognition. The SSL-based model utilized learned biometric representations to effectively detect drug use patterns without explicit labels. Personalized models mitigate inter-person overfitting more effectively than a single model designed for all participants by training individually on data specific to each person. We implemented both supervised and SSL-based models using TensorFlow version 2.17.0. [39].

#### 3.4.1. Convolutional Neural Network Architectures

We implemented a 1D CNN for binary classification of time-series data. Initially, we fed selected features from each participant as input into the 1D CNN. This network consisted of a convolutional layer equipped with 32 size-3 filters using ReLU activation for nonlinear processing. Following this layer, we implemented a max pooling layer with a pool size of 2, which effectively reduced the dimensionality of the feature maps from 10 to 5 per channel. We then flattened the pooled outputs into a 160-unit vector and applied a dropout layer with a 50% rate [40] to prevent overfitting. The network ended with a dense layer with sigmoid activation, outputting the drug use probability. Optimized with the Brier score, this efficient architecture ensured effective learning from time-series data.

#### 3.4.2. Supervised Learning Framework

**Figure 3.**
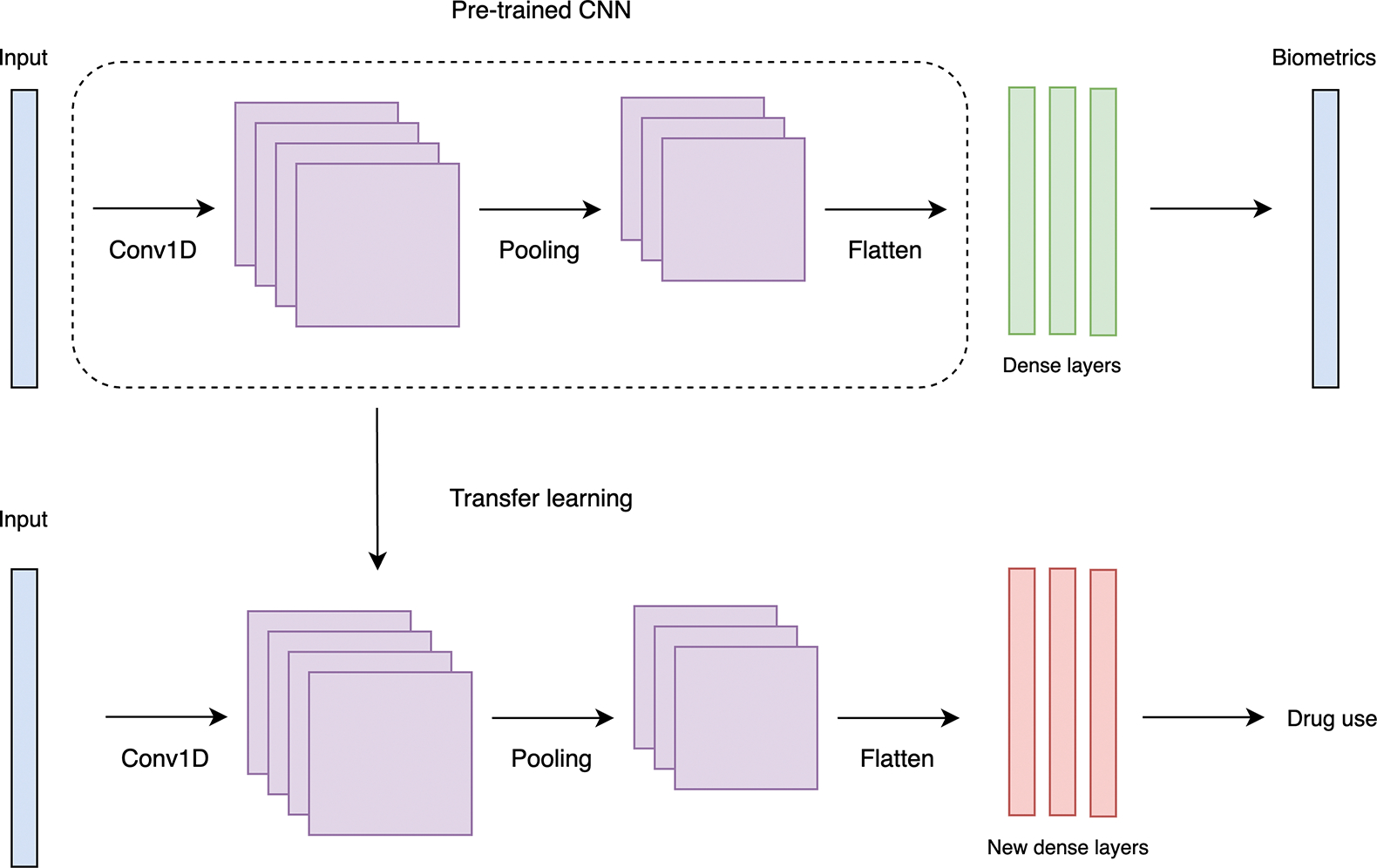
An SSL-enhanced transfer learning framework for drug use classification, utilizing selected biometric features from each participant. A CNN pre-trained with SSL, outlined with a dotted line around the 1D convulutional, pooling, and flatten layers, is fine-tuned with new dense layers to predict drug use from biometric featrues. The dotted line indicates the layers transferred for the task-specific model.

The supervised learning framework used a pre-trained model for multi-output regression on biometric data and transfer learning for drug use classification, as depicted in Figure 3. In the pre-training phase, the model utilized identical hyperparameters to the supervised CNN to maintain consistency. It processed selected features from each participant using a Conv1D layer for feature extraction, a pooling layer to reduce dimensions, and a flatten layer that transforms feature maps into vectors. We then passed these vectors through dense layers and used mean squared error for biometric predictions. Subsequently, we used the weights from the convolutional, pooling, and flatten layers for fine-tuning. We augmented this backbone with new dense layers specifically designed for the binary classification of drug use and fine-tuned the model to this new task. Additions included a dense layer with 32 units and ReLU activation, a dropout layer with a 50% rate to mitigate overfitting, and a final dense layer with sigmoid activation for binary output. The fine-tuning phase similarly used the Brier score for optimization.

#### 3.4.3. Optimization and Training

The base CNN employed the Adam optimizer [41] with a 0.001 initial learning rate and an early stopping criterion of up to 10 epochs, with ReduceLROnPlateau [39] used as a callback algorithm. We trained the model for 200 epochs, with a batch size of 32 and a 20% validation split.

For the SSL framework, the pre-training phase involved training the CNN model for 100 epochs on biometric features. After the weights were transferred, we froze the transferred layers for the first 100 epochs of training. We then unfroze these layers for an additional 100 epochs of training, with the transferred model using a batch size of 32 and a 20% validation split for fine-tuning.

### 3.5. Experiments

We aimed to continuously predict drug use among participants (binary classification) within 1 h windows, based on processed features. To evaluate the model, we used a 70–30 split for training and testing the data, corresponding to the monitoring period. This split ensured that the test data included a sufficient representation of drug use instances for evaluation. We independently trained and tested the models using data from each participant to achieve personalization. This approach enabled us to tailor the predictive models to individual behavioral patterns, potentially enhancing the accuracy and relevance of the predictions for each subject.

### 3.6. Evaluation

To assess the performance of our models, we focused on four metrics: sensitivity, specificity, precision, and the area under the receiver operating characteristic curve (AUC), defined as follows:

(1) $$\text{Sensitivity}(TPR)=\frac{TP}{TP\;+\;FN}$$

(2) $$\text{Specificity}(TNR)=\frac{TN}{FP\;+\;TN}$$

(3) $$\text{Precision}(PPV)=\frac{TP}{TP\;+\;FP}$$

(4) AUC=AreaundertheROCcurve

Sensitivity, also known as the true positive rate (TPR), is crucial for identifying all relevant cases of drug use, ensuring that no positive instance is missed. Specificity, or true negative rate (TNR), is vital for reducing false alarms by correctly identifying non-use instances, which helps in avoiding unnecessary interventions. Precision, the positive predictive value (PPV), measures the reliability of the model in predicting positive outcomes, indicating the probability that a predicted positive is an actual case of drug use. The AUC measures the overall ability of the model to discriminate between positive (drug use) and negative (non-use) classes across all possible classification thresholds. These metrics collectively assess the model’s effectiveness in correctly identifying and classifying drug use instances. We used these four metrics to evaluate the personalized model developed for each participant using only their data.

To explore the effect of classification thresholds, we extracted predictions from the emitted probabilities of our models using thresholds ranging from 0.1 to 0.9, incremented by 0.1. To evaluate the robustness of the models, we used block bootstrapping on the test data specific to each participant. We applied the block bootstrap for 30 trials and recorded the means and standard deviations of the evaluation metrics. This method selects contiguous blocks of data, sized according to the square root of the test set length, to preserve the sequence integrity of the dataset. This is particularly important for time-series models, as it helps to ensure that the performance metrics accurately reflect realistic conditions. In contrast to cross-validation, block bootstrapping also addresses the issue where the infrequency of drug use events may result in many folds having test sets lacking any drug use labels.

## 4. Results

Table 1 presents a summary of the data quality for all participants, quantifying the percentage of device-wearing time (HR data coverage) per participant, the total duration of the monitoring period based on the recorded HR data in days, and the number of drug use instances.

Table 2 presents the performance metrics for both purely supervised and self-supervised learning models developed for each participant, highlighting variability in specificity and sensitivity at the 0.5 decision threshold as well as AUC, along with the specific substances used by each participant. Among the 9 participants, we achieved an average AUC of 0.695 for the supervised CNN and 0.729 for the SSL model. We observe that for most participants, SSL results in a higher AUC than the supervised CNN, except for participants 12 and 27, where the supervised model performed slightly better. At a threshold of 0.5, both supervised learning and SSL tend to have higher specificity compared to the corresponding sensitivity, indicating a stronger ability to identify true negatives. Notably, there is an increase in sensitivity with SSL for most participants. In addition, we find that there is no significant correlation between our data quality summary metrics and model performance, although we note that our summary metrics do not capture the intricacies of the data collection process.

Tables 3–5 display the test performances for participants 14, 18, and 20 at various decision thresholds. We highlighted these participants due to their relatively consistent high AUC at a 0.5 decision threshold, indicating high-quality data from these participants. Supplementary Material is available for the test results of all participants across all thresholds. We observe that participants 14, 18, and 20 not only achieved higher AUC values but also maintained more stable performance metrics in terms of specificity and sensitivity when varying the decision threshold compared to other participants, providing another indicator of high-quality data.

**Figure 4.**
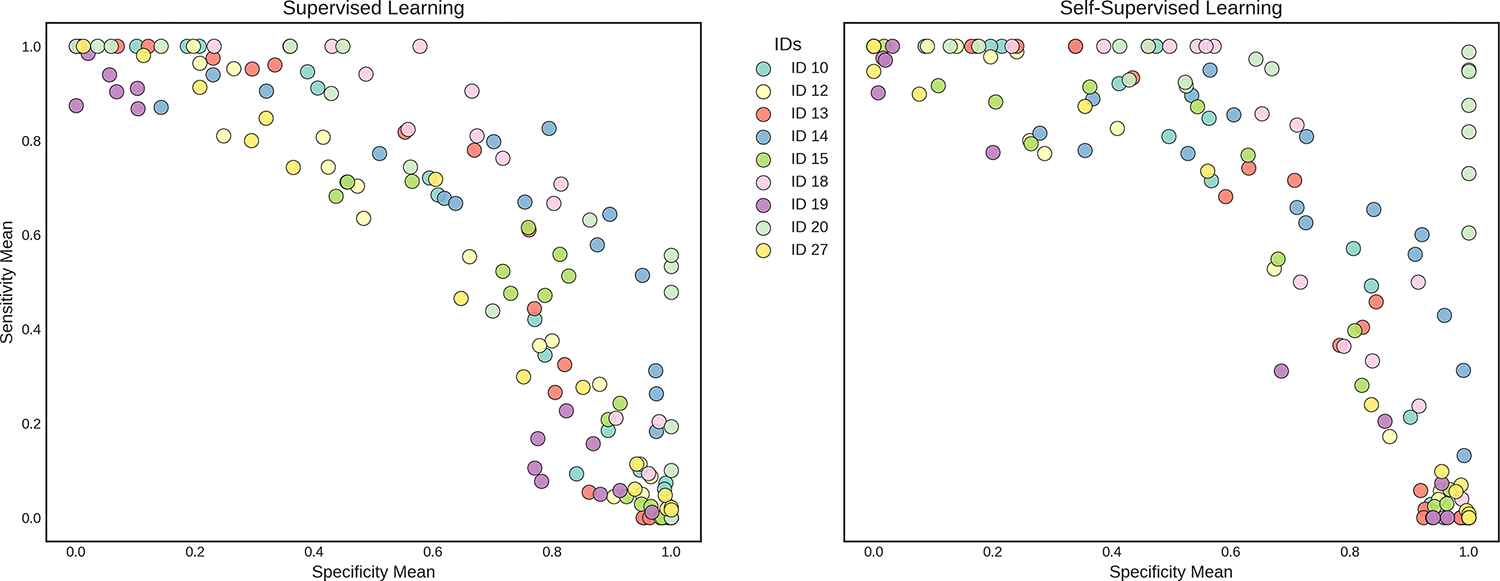
Mean bootstrapped sensitivity and specificity at different decision threshold cutoffs across 9 participants, each denoted by distinct colors.

Figure 4 illustrates the trade-offs between the mean sensitivity and specificity across different thresholds for participants 14 and 18, as detailed in Table 6. We specifically chose these participants for this analysis due to their higher AUC values and the stability of their performance metrics across various thresholds. When selecting thresholds, our strategy prioritized achieving high specificity while ensuring reasonable sensitivity, typically around 0.5, and similarly, high sensitivity while maintaining acceptable specificity levels, based on the training performance. We observe from the table that, for Participant 14, the threshold of 0.31 results in a high sensitivity of 0.969 with a modest specificity of 0.543, which suits scenarios where false negatives are more critical to avoid. Conversely, for Participant 18, a threshold of 0.66 yields a specificity of 0.915 and a sensitivity of 0.516, demonstrating a scenario where reducing false positives is prioritized.

## 5. Discussion

### 5.1. Implications

With modest AUC scores, we were able to choose thresholds that minimize either false positives or false negatives. For example, for most participants, we were able to choose a threshold with low sensitivity but high specificity, minimizing false positives. This approach is beneficial in contexts where it is essential to minimize false alarms, such as in contexts where the triggered intervention would be costly to the healthcare provider. By contrast, when setting a threshold that favors high sensitivity with low specificity, the model effectively detects most potential drug use instances, albeit at the cost of more false positives. This configuration can ensure that most if not all drug use cases are detected, which is useful for low-cost interventions that can be deployed even when a participant is not necessarily using drugs. The choice of an ideal threshold depends on the desired translational use case.

Overall, the SSL models tended to reach slightly higher average AUC scores than their supervised equivalents, though the improvements in sensitivity and specificity were not consistent across participants and across model thresholds. Therefore, we consider the evaluation of personalized SSL to have essentially yielded a negative result. This might be due to low-quality data resulting from participant non-compliance [42], as often occurred in our study. For example, lifestyle changes and privacy concerns led ID 12 to participate inconsistently and omit frequent EMAs, which compromised the reliability of data collection. Conversely, ID 19 and 20, who are both graduate students, demonstrated the highest degree of performance improvements with the SSL model over the baseline model. These qualitative observations reinforce the importance of data collection quality and suggest that more thorough procedures are needed in future work to ensure participant compliance. In addition, we need more data to thoroughly evaluate the SSL model using quantitative measures of data quality and model performance.

Additionally, the evaluation results exhibit high standard deviations, pointing to significant variability in the models’ performance across different time segments. This inconsistency in the models’ performance across the test data highlights the challenge in working with real-world biosensor data from a study population using substances.

### 5.2. Future Steps and Limitations

Our study has some notable limitations. One significant limitation is the relatively small sample size, which may limit the generalizability of our findings to broader populations. This constraint could result in overfitting, where the model performs well on the specific dataset used for training and testing but may struggle to maintain its accuracy across diverse or larger populations. The small data sample also restricts our ability to use more robust model architectures, such as self-attention-based models, since complex models with more parameters are likely to overfit a small dataset. To address this limitation in future work, we will collect more data from more participants and conduct a more comprehensive examination using quantitative measures of data quality and model performance. Furthermore, the models’ performance exhibited high within-person variation, indicating the need for more data for each participant and noise-reducing data collection procedures. Another major limitation is the frequent occurrence of low-quality data collection due to participant non-compliance. To compound this issue, Fitbit can introduce measurement errors, which may limit the accuracy of the collected biometric data. Additionally, certain substances are associated with an increase in HR, while others may lower it, leading to physiological states that differ significantly from the baseline. Furthermore, the interaction between different substances could compound or alter these effects, adding complexity to HR monitoring. This variation and interaction in HR response could significantly influence the model’s ability to detect drug use consistently across different substances, potentially leading to discrepancies in detection accuracy. Such variability highlights the importance of developing models that can adapt to or account for the diverse and interacting impacts of various substances on HR to ensure robust and reliable performance across different contexts.

## Supplementary Material

Supplementary Material

## Figures and Tables

**Table 1.** Data quality summary metrics for each of the 9 study participants.

ID	Drug Use Instances	Drugs Used	Wearing Time	Monitoring Period (Days)

27	197	2	73.70%	27
20	31	2	33.14%	6
19	63	3	68.94%	28
18	38	1	97.99%	29
15	59	2	95.72%	29
14	63	1	89.61%	29
13	104	3	60.40%	27
12	74	4	85.09%	27
10	52	3	90.87%	27

**Table 2.** Performance metrics for supervised and self-supervised learning. Bold values in the AUC column indicate higher performance.

Participants ID	Supervised Learning			Self-Supervised Learning			Substance Used
	
	Specificity	Sensitivity	AUC	Specificity	Sensitivity	AUC	

10	0.788	0.474	0.687	0.657	0.700	**0.758**	Alcohol, Cannabis, Nicotine
12	0.661	0.563	**0.659**	0.668	0.536	0.642	Alcohol, Meth, Nicotine, GHB
13	0.772	0.527	0.703	0.742	0.530	**0.714**	Alcohol, Cannabis, Nicotine
14	0.768	0.809	0.744	0.693	0.883	**0.753**	Cannabis
15	0.820	0.428	0.662	0.777	0.543	**0.667**	Cannabis, Mushroom
18	0.703	0.824	0.808	0.727	0.632	**0.834**	Cannabis
19	0.769	0.199	0.478	0.760	0.206	**0.601**	Meth, Alcohol
20	0.478	0.649	0.879	0.456	0.935	**0.980**	Meth, Nicotine
27	0.658	0.491	**0.638**	0.758	0.349	0.613	Meth, Nicotine

**Table 3.** Comparison of specificity and sensitivity by decision threshold for CNN and SSL models for ID 14.

Threshold	Supervised Learning (CNN)		Self-Supervised Learning (SSL)	
	
	Specificity (Mean ± SD)	Sensitivity (Mean ± SD)	Specificity (Mean ± SD)	Sensitivity (Mean ± SD)

0.1	0.203 ± 0.135	0.820 ± 0.322	0.343 ± 0.191	0.875 ± 0.258
0.2	0.346 ± 0.185	0.945 ± 0.210	0.360 ± 0.214	0.917 ± 0.234
0.3	0.485 ± 0.163	0.883 ± 0.271	0.498 ± 0.151	0.904 ± 0.190
0.4	0.588 ± 0.174	0.68 ± 0.434	0.593 ± 0.199	0.799 ± 0.290
0.5	0.768 ± 0.091	0.809 ± 0.304	0.693 ± 0.146	0.883 ± 0.205
0.6	0.833 ± 0.118	0.619 ± 0.415	0.851 ± 0.136	0.713 ± 0.396
0.7	0.923 ± 0.068	0.458 ± 0.401	0.921 ± 0.094	0.569 ± 0.399
0.8	0.978 ± 0.043	0.311 ± 0.412	0.983 ± 0.045	0.381 ± 0.396
0.9	1.000 ± 0.000	0.167 ±0.267	1.000 ± 0.000	0.115 ± 0.252

**Table 4.** Comparison of specificity and sensitivity by decision threshold for CNN and SSL models for ID 18.

Threshold	Supervised Learning		Self-Supervised Learning	
	
	Specificity (Mean ± SD)	Sensitivity (Mean ± SD)	Specificity (Mean ± SD)	Sensitivity (Mean ± SD)

0.1	0.245 ± 0.249	1.000 ± 0.000	0.185 ± 0.195	1.000 ± 0.000
0.2	0.365 ± 0.188	1.000 ± 0.000	0.440 ± 0.214	1.000 ± 0.000
0.3	0.482 ± 0.147	0.882 ± 0.322	0.628 ± 0.187	1.000 ± 0.000
0.4	0.637 ± 0.139	0.826 ± 0.379	0.715 ± 0.133	0.880 ± 0.214
0.5	0.703 ± 0.131	0.824 ± 0.340	0.727 ± 0.140	0.632 ± 0.392
0.6	0.863 ± 0.141	0.583 ± 0.373	0.855 ± 0.149	0.447 ± 0.484
0.7	0.943 ± 0.060	0.083 ± 0.186	0.932 ± 0.085	0.350 ± 0.391
0.8	0.989 ± 0.027	0.071 ± 0.175	1.000 ± 0.000	0.000 ± 0.000
0.9	1.000 ± 0.000	0.000 ± 0.000	1.000 ± 0.000	0.000 ± 0.000

**Table 5.** Comparison of specificity and sensitivity by decision threshold for CNN and SSL models for ID 20.

Threshold	Supervised Learning (CNN)		Self-Supervised Learning (SSL)	
	
	Specificity (Mean ± SD)	Sensitivity (Mean ± SD)	Specificity (Mean ± SD)	Sensitivity (Mean ± SD)

0.1	0.000 ± 0.000	1.000 ± 0.000	0.085 ± 0.143	1.000 ± 0.000
0.2	0.042 ± 0.161	1.000 ± 0.000	0.330 ± 0.414	1.000 ± 0.000
0.3	0.295 ± 0.397	1.000 ± 0.000	0.457 ± 0.403	0.931 ± 0.117
0.4	0.583 ± 0.470	0.920 ± 0.098	0.713 ± 0.408	0.933 ± 0.128
0.5	0.478 ± 0.500	0.649 ± 0.216	0.456 ± 0.458	0.935 ± 0.111
0.6	1.000 ± 0.000	0.675 ± 0.263	1.000 ± 0.000	0.947 ± 0.112
0.7	1.000 ± 0.000	0.405 ± 0.355	1.000 ± 0.000	0.903 ± 0.110
0.8	1.000 ± 0.000	0.260 ± 0.238	1.000 ± 0.000	0.756 ± 0.172
0.9	1.000 ± 0.000	0.000 ± 0.000	1.000 ± 0.000	0.597 ± 0.085

**Table 6.** Specificity and sensitivity tradeoff for participants 14 and 18. Bold values indicate prioritized metric for each threshold.

Participants ID	Threshold	Train Performance		Test Performance		Model Type
	
		Specificity	Sensitivity	Specificity Mean	Sensitivity Mean	

18	0.63	**0.863**	0.700	**0.873 ± 0.115**	0.55 ± 0.384	CNN
18	0.66	**0.915**	0.516	**0.915 ± 0.071**	0.5 ± 0.5	SSL
18	0.36	0.579	**0.974**	0.578 ± 0.176	**1**	CNN
18	0.36	0.614	**0.946**	0.558 ± 0.214	**1**	SSL
14	0.66	**0.904**	0.539	**0.897 ± 0.086**	0.644 ± 0.387	CNN
14	0.65	**0.926**	0.625	**0.901 ± 0.092**	0.636 ± 0.322	SSL
14	0.31	0.499	**0.980**	0.506 ± 0.156	**0.821 ± 0.361**	CNN
14	0.31	0.543	**0.969**	0.534 ± 0.171	**0.896 ± 0.248**	SSL

## Data Availability

Fully de-identified raw data are available upon request.
