# Supplementary material for: Monitoring Substance Use with Fitbit Biosignals: A Case Study on Training Deep Learning Models Using Ecological Momentary Assessments and Passive Sensing
Source: AI (Basel). Author manuscript; Available in PMC 2025 May 11. (PMC12065672; doi:10.3390/ai5040131)
Supplement: Supplementary Material [file NIHMS2074212-supplement-Supplementary_Material.pdf]

Supplementary Information: Results of All Participants

ID 10

Table S1: Evaluation metrics by frequency and threshold of CNN control model for ID 10. The AUC score is 0.687.

Threshold	Specificity		Sensitivity		Precision		F1 Score	
	Mean	SD	Mean	SD	Mean	SD	Mean	SD
0.1	0.062	0.073	1.000	0.000	0.204	0.066	0.334	0.093
0.11	0.102	0.093	1.000	0.000	0.197	0.069	0.324	0.096
0.12	0.112	0.086	1.000	0.000	0.186	0.074	0.307	0.106
0.13	0.151	0.120	1.000	0.000	0.186	0.067	0.308	0.093
0.14	0.177	0.147	1.000	0.000	0.207	0.083	0.335	0.110
0.15	0.141	0.121	1.000	0.000	0.213	0.073	0.345	0.099
0.16	0.187	0.176	1.000	0.000	0.217	0.065	0.351	0.089
0.17	0.215	0.170	1.000	0.000	0.215	0.063	0.350	0.087
0.18	0.258	0.141	1.000	0.000	0.196	0.068	0.322	0.092
0.19	0.236	0.176	1.000	0.000	0.210	0.058	0.344	0.079
0.2	0.278	0.140	1.000	0.000	0.242	0.069	0.385	0.092
0.21	0.207	0.158	1.000	0.000	0.232	0.078	0.371	0.101
0.22	0.231	0.195	1.000	0.000	0.224	0.089	0.357	0.123
0.23	0.304	0.186	1.000	0.000	0.220	0.081	0.353	0.112
0.24	0.250	0.168	1.000	0.000	0.241	0.073	0.382	0.099
0.25	0.397	0.205	1.000	0.000	0.279	0.107	0.426	0.132
0.26	0.406	0.155	0.912	0.143	0.245	0.066	0.377	0.080
0.27	0.379	0.162	0.972	0.079	0.232	0.084	0.365	0.098
0.28	0.368	0.175	0.921	0.145	0.243	0.075	0.373	0.086
0.29	0.440	0.191	0.917	0.175	0.269	0.089	0.404	0.107
0.3	0.480	0.219	0.769	0.277	0.246	0.136	0.357	0.170
0.31	0.389	0.204	0.946	0.127	0.282	0.114	0.420	0.136
0.32	0.433	0.242	0.897	0.189	0.252	0.133	0.376	0.152
0.33	0.489	0.217	0.824	0.239	0.240	0.125	0.356	0.160
0.34	0.432	0.185	0.811	0.281	0.211	0.101	0.324	0.142
0.35	0.546	0.203	0.671	0.363	0.184	0.116	0.278	0.159
0.36	0.594	0.190	0.721	0.284	0.244	0.112	0.358	0.151
0.37	0.595	0.191	0.537	0.391	0.221	0.178	0.292	0.217
0.38	0.581	0.185	0.615	0.370	0.195	0.111	0.289	0.163
0.39	0.570	0.186	0.704	0.375	0.244	0.147	0.343	0.184
0.4	0.580	0.249	0.745	0.334	0.261	0.157	0.361	0.188
0.41	0.608	0.181	0.685	0.425	0.238	0.176	0.339	0.227
0.42	0.681	0.186	0.496	0.360	0.261	0.201	0.318	0.229
0.43	0.612	0.215	0.485	0.395	0.176	0.143	0.237	0.179
0.44	0.593	0.248	0.562	0.380	0.253	0.200	0.318	0.232
0.45	0.773	0.175	0.361	0.266	0.297	0.221	0.306	0.228
0.46	0.788	0.086	0.345	0.365	0.231	0.251	0.256	0.268
0.47	0.766	0.195	0.377	0.357	0.253	0.240	0.286	0.271
0.48	0.746	0.185	0.325	0.306	0.292	0.336	0.284	0.307
0.49	0.758	0.197	0.432	0.396	0.284	0.245	0.293	0.249
0.5	0.788	0.124	0.474	0.405	0.327	0.319	0.317	0.246
0.51	0.771	0.186	0.421	0.363	0.336	0.339	0.317	0.278
0.52	0.847	0.110	0.358	0.366	0.308	0.322	0.304	0.297
0.53	0.845	0.157	0.363	0.293	0.339	0.338	0.319	0.268
0.54	0.879	0.164	0.230	0.249	0.279	0.369	0.225	0.260
0.55	0.891	0.121	0.157	0.246	0.103	0.175	0.121	0.197
0.56	0.841	0.155	0.093	0.210	0.111	0.248	0.101	0.226

Continued on next page

Threshold	Specificity		Sensitivity		Precision		F1 Score	
	Mean	SD	Mean	SD	Mean	SD	Mean	SD
0.57	0.914	0.138	0.040	0.143	0.072	0.240	0.046	0.155
0.58	0.934	0.079	0.189	0.310	0.254	0.391	0.195	0.296
0.59	0.914	0.119	0.154	0.294	0.200	0.356	0.160	0.285
0.6	0.944	0.094	0.139	0.240	0.238	0.387	0.162	0.262
0.61	0.894	0.124	0.185	0.325	0.202	0.334	0.171	0.278
0.62	0.936	0.079	0.176	0.295	0.269	0.397	0.183	0.269
0.63	0.976	0.058	0.131	0.235	0.286	0.452	0.171	0.291
0.64	0.964	0.087	0.048	0.172	0.071	0.258	0.057	0.206
0.65	0.922	0.100	0.069	0.172	0.167	0.373	0.094	0.222
0.66	0.948	0.090	0.101	0.214	0.211	0.408	0.132	0.268
0.67	0.947	0.077	0.044	0.131	0.105	0.307	0.061	0.181
0.68	0.971	0.064	0.127	0.162	0.412	0.492	0.192	0.237
0.69	0.926	0.094	0.074	0.134	0.235	0.424	0.112	0.203
0.7	0.949	0.084	0.148	0.192	0.389	0.487	0.213	0.271
0.71	0.956	0.072	0.000	0.000	0.000	0.000	0.000	0.000
0.72	0.963	0.069	0.074	0.169	0.167	0.373	0.102	0.230
0.73	0.983	0.033	0.053	0.122	0.158	0.365	0.079	0.182
0.74	0.939	0.065	0.061	0.145	0.158	0.365	0.088	0.205
0.75	0.963	0.066	0.058	0.142	0.150	0.357	0.083	0.201
0.76	0.991	0.026	0.074	0.139	0.222	0.416	0.111	0.208
0.77	0.974	0.039	0.061	0.145	0.158	0.365	0.088	0.205
0.78	0.990	0.027	0.039	0.107	0.118	0.322	0.059	0.161
0.79	0.986	0.040	0.065	0.145	0.174	0.379	0.094	0.208
0.8	0.974	0.050	0.026	0.089	0.077	0.266	0.038	0.133
0.81	0.989	0.029	0.061	0.129	0.182	0.386	0.091	0.193
0.82	0.979	0.052	0.000	0.000	0.000	0.000	0.000	0.000
0.83	0.973	0.061	0.000	0.000	0.000	0.000	0.000	0.000
0.84	0.995	0.019	0.000	0.000	0.000	0.000	0.000	0.000
0.85	0.989	0.028	0.000	0.000	0.000	0.000	0.000	0.000
0.86	0.995	0.019	0.000	0.000	0.000	0.000	0.000	0.000
0.87	1.000	0.000	0.000	0.000	0.000	0.000	0.000	0.000
0.88	1.000	0.000	0.000	0.000	0.000	0.000	0.000	0.000
0.89	1.000	0.000	0.000	0.000	0.000	0.000	0.000	0.000
0.9	1.000	0.000	0.000	0.000	0.000	0.000	0.000	0.000

Table S2: Evaluation metrics by frequency and threshold of CNN SSL model for ID 10. The AUC score is 0.758.

Threshold	Specificity		Sensitivity		Precision		F1 Score	
	Mean	SD	Mean	SD	Mean	SD	Mean	SD
0.1	0.031	0.077	1.000	0.000	0.185	0.057	0.309	0.078
0.11	0.086	0.108	1.000	0.000	0.200	0.086	0.324	0.118
0.12	0.058	0.105	1.000	0.000	0.187	0.067	0.310	0.095
0.13	0.119	0.124	1.000	0.000	0.194	0.069	0.319	0.093
0.14	0.122	0.115	1.000	0.000	0.189	0.054	0.315	0.075
0.15	0.184	0.166	1.000	0.000	0.210	0.068	0.343	0.091
0.16	0.215	0.157	1.000	0.000	0.213	0.072	0.345	0.098
0.17	0.233	0.184	1.000	0.000	0.225	0.079	0.360	0.107
0.18	0.242	0.187	1.000	0.000	0.206	0.073	0.335	0.098
0.19	0.271	0.205	1.000	0.000	0.214	0.080	0.345	0.106
0.2	0.201	0.181	1.000	0.000	0.209	0.095	0.336	0.128
0.21	0.196	0.128	1.000	0.000	0.186	0.078	0.307	0.106
0.22	0.207	0.167	1.000	0.000	0.236	0.087	0.374	0.111
0.23	0.221	0.173	1.000	0.000	0.253	0.089	0.396	0.113
0.24	0.314	0.242	1.000	0.000	0.244	0.091	0.384	0.116
0.25	0.307	0.229	1.000	0.000	0.236	0.083	0.374	0.107
0.26	0.339	0.184	1.000	0.000	0.253	0.100	0.394	0.131
0.27	0.307	0.171	1.000	0.000	0.239	0.096	0.376	0.128
0.28	0.368	0.154	1.000	0.000	0.247	0.099	0.387	0.127
0.29	0.362	0.173	1.000	0.000	0.255	0.082	0.400	0.102
0.3	0.385	0.195	1.000	0.000	0.227	0.095	0.360	0.123
0.31	0.474	0.132	1.000	0.000	0.320	0.082	0.479	0.095
0.32	0.374	0.199	1.000	0.000	0.249	0.099	0.389	0.127
0.33	0.438	0.184	1.000	0.000	0.279	0.102	0.427	0.128
0.34	0.399	0.186	0.932	0.172	0.291	0.112	0.436	0.143
0.35	0.408	0.219	0.859	0.216	0.219	0.086	0.342	0.116
0.36	0.412	0.163	0.921	0.182	0.225	0.074	0.358	0.103
0.37	0.405	0.194	0.875	0.199	0.246	0.110	0.372	0.135
0.38	0.463	0.169	0.882	0.194	0.277	0.122	0.409	0.148
0.39	0.507	0.192	0.892	0.178	0.315	0.128	0.453	0.153
0.4	0.402	0.158	0.932	0.154	0.266	0.098	0.404	0.124
0.41	0.496	0.212	0.809	0.291	0.277	0.156	0.402	0.199
0.42	0.443	0.137	0.881	0.198	0.270	0.120	0.402	0.138
0.43	0.498	0.191	0.869	0.213	0.303	0.156	0.435	0.177
0.44	0.520	0.196	0.896	0.188	0.287	0.106	0.424	0.132
0.45	0.618	0.202	0.929	0.157	0.388	0.190	0.524	0.180
0.46	0.563	0.225	0.848	0.273	0.331	0.201	0.426	0.157
0.47	0.556	0.162	0.833	0.276	0.294	0.110	0.420	0.137
0.48	0.660	0.206	0.816	0.370	0.347	0.237	0.464	0.260
0.49	0.600	0.240	0.762	0.340	0.350	0.208	0.444	0.224
0.5	0.657	0.233	0.700	0.392	0.344	0.278	0.406	0.260
0.51	0.567	0.237	0.715	0.360	0.306	0.242	0.386	0.250
0.52	0.645	0.210	0.625	0.363	0.286	0.165	0.367	0.197
0.53	0.673	0.174	0.600	0.490	0.250	0.249	0.338	0.312
0.54	0.686	0.196	0.630	0.448	0.268	0.230	0.363	0.290
0.55	0.704	0.166	0.476	0.449	0.169	0.174	0.244	0.243
0.56	0.806	0.143	0.571	0.444	0.353	0.331	0.400	0.315
0.57	0.812	0.149	0.475	0.487	0.235	0.285	0.303	0.336
0.58	0.757	0.115	0.548	0.486	0.298	0.291	0.367	0.327
0.59	0.849	0.114	0.350	0.450	0.204	0.301	0.234	0.314
0.6	0.835	0.130	0.333	0.429	0.251	0.322	0.275	0.341
0.61	0.836	0.112	0.492	0.476	0.328	0.328	0.389	0.378
0.62	0.829	0.094	0.333	0.424	0.231	0.303	0.268	0.340

Continued on next page

Threshold	Specificity		Sensitivity		Precision		F1 Score	
	Mean	SD	Mean	SD	Mean	SD	Mean	SD
0.63	0.886	0.110	0.343	0.383	0.274	0.319	0.294	0.324
0.64	0.903	0.107	0.265	0.404	0.192	0.321	0.214	0.340
0.65	0.908	0.084	0.333	0.422	0.247	0.330	0.278	0.360
0.66	0.902	0.083	0.213	0.335	0.244	0.349	0.219	0.323
0.67	0.936	0.067	0.039	0.107	0.059	0.161	0.047	0.129
0.68	0.943	0.074	0.315	0.335	0.444	0.437	0.363	0.370
0.69	0.928	0.086	0.147	0.208	0.269	0.398	0.190	0.272
0.7	0.961	0.059	0.056	0.112	0.200	0.400	0.087	0.175
0.71	0.936	0.094	0.028	0.079	0.111	0.314	0.044	0.126
0.72	0.946	0.087	0.036	0.095	0.130	0.337	0.057	0.147
0.73	0.948	0.077	0.013	0.056	0.053	0.223	0.021	0.089
0.74	0.971	0.051	0.000	0.000	0.000	0.000	0.000	0.000
0.75	0.959	0.063	0.000	0.000	0.000	0.000	0.000	0.000
0.76	0.995	0.019	0.000	0.000	0.000	0.000	0.000	0.000
0.77	0.990	0.035	0.000	0.000	0.000	0.000	0.000	0.000
0.78	0.983	0.042	0.000	0.000	0.000	0.000	0.000	0.000
0.79	0.991	0.037	0.000	0.000	0.000	0.000	0.000	0.000
0.8	1.000	0.000	0.000	0.000	0.000	0.000	0.000	0.000
0.81	0.995	0.020	0.000	0.000	0.000	0.000	0.000	0.000
0.82	1.000	0.000	0.000	0.000	0.000	0.000	0.000	0.000
0.83	1.000	0.000	0.000	0.000	0.000	0.000	0.000	0.000
0.84	1.000	0.000	0.000	0.000	0.000	0.000	0.000	0.000
0.85	1.000	0.000	0.000	0.000	0.000	0.000	0.000	0.000
0.86	1.000	0.000	0.000	0.000	0.000	0.000	0.000	0.000
0.87	1.000	0.000	0.000	0.000	0.000	0.000	0.000	0.000
0.88	1.000	0.000	0.000	0.000	0.000	0.000	0.000	0.000
0.89	1.000	0.000	0.000	0.000	0.000	0.000	0.000	0.000
0.9	1.000	0.000	0.000	0.000	0.000	0.000	0.000	0.000

ID 12

Table S3: Evaluation metrics by frequency and threshold of CNN control model for ID 12. The AUC score is 0.659.

Threshold	Specificity		Sensitivity		Precision		F1 Score	
	Mean	SD	Mean	SD	Mean	SD	Mean	SD
0.1	0.195	0.191	1.000	0.000	0.370	0.204	0.509	0.212
0.11	0.197	0.218	1.000	0.000	0.428	0.194	0.572	0.205
0.12	0.178	0.201	0.976	0.106	0.361	0.096	0.522	0.112
0.13	0.199	0.216	1.000	0.000	0.399	0.127	0.559	0.125
0.14	0.175	0.229	1.000	0.000	0.509	0.225	0.643	0.216
0.15	0.160	0.187	0.982	0.074	0.392	0.168	0.541	0.180
0.16	0.265	0.240	0.953	0.098	0.350	0.128	0.499	0.140
0.17	0.234	0.218	0.935	0.132	0.433	0.173	0.578	0.171
0.18	0.334	0.248	0.937	0.115	0.371	0.133	0.519	0.154
0.19	0.261	0.265	0.984	0.071	0.438	0.221	0.574	0.222
0.2	0.188	0.246	0.967	0.145	0.358	0.135	0.510	0.162
0.21	0.208	0.216	0.964	0.071	0.409	0.205	0.545	0.205
0.22	0.415	0.265	0.815	0.307	0.423	0.244	0.536	0.268
0.23	0.198	0.213	0.806	0.256	0.391	0.199	0.511	0.210
0.24	0.401	0.288	0.799	0.307	0.415	0.216	0.534	0.235
0.25	0.338	0.301	0.874	0.242	0.467	0.250	0.585	0.256
0.26	0.248	0.232	0.810	0.261	0.367	0.214	0.485	0.225
0.27	0.369	0.339	0.943	0.095	0.435	0.184	0.575	0.179
0.28	0.334	0.285	0.777	0.306	0.429	0.257	0.536	0.270
0.29	0.348	0.320	0.936	0.109	0.448	0.230	0.572	0.208
0.3	0.415	0.328	0.911	0.126	0.483	0.214	0.610	0.193
0.31	0.415	0.294	0.807	0.299	0.412	0.255	0.517	0.268
0.32	0.307	0.295	0.869	0.174	0.419	0.226	0.536	0.223
0.33	0.449	0.319	0.910	0.178	0.481	0.234	0.598	0.223
0.34	0.500	0.251	0.855	0.219	0.417	0.174	0.528	0.171
0.35	0.374	0.322	0.673	0.333	0.405	0.269	0.484	0.287
0.36	0.424	0.264	0.744	0.310	0.369	0.169	0.478	0.204
0.37	0.458	0.249	0.661	0.352	0.400	0.244	0.484	0.276
0.38	0.502	0.306	0.754	0.326	0.465	0.268	0.544	0.275
0.39	0.411	0.296	0.666	0.330	0.435	0.297	0.512	0.303
0.4	0.517	0.288	0.659	0.323	0.437	0.288	0.503	0.291
0.41	0.473	0.324	0.704	0.283	0.464	0.267	0.523	0.256
0.42	0.562	0.269	0.787	0.235	0.508	0.248	0.602	0.231
0.43	0.549	0.226	0.653	0.306	0.409	0.197	0.470	0.204
0.44	0.624	0.232	0.624	0.317	0.459	0.276	0.510	0.279
0.45	0.654	0.283	0.717	0.286	0.537	0.316	0.586	0.285
0.46	0.483	0.243	0.635	0.279	0.432	0.225	0.490	0.228
0.47	0.637	0.322	0.635	0.274	0.491	0.302	0.506	0.246
0.48	0.642	0.265	0.643	0.351	0.432	0.243	0.475	0.261
0.49	0.551	0.225	0.531	0.323	0.389	0.265	0.439	0.279
0.5	0.661	0.255	0.563	0.368	0.396	0.313	0.411	0.273
0.51	0.661	0.278	0.554	0.247	0.559	0.313	0.490	0.199
0.52	0.759	0.202	0.379	0.297	0.403	0.260	0.372	0.264
0.53	0.609	0.227	0.463	0.396	0.304	0.256	0.307	0.220
0.54	0.746	0.189	0.441	0.255	0.525	0.211	0.451	0.205
0.55	0.696	0.344	0.496	0.302	0.515	0.323	0.450	0.250
0.56	0.800	0.192	0.375	0.305	0.486	0.371	0.383	0.279
0.57	0.711	0.250	0.428	0.278	0.458	0.293	0.411	0.246
0.58	0.764	0.184	0.428	0.334	0.397	0.288	0.376	0.249
0.59	0.811	0.173	0.290	0.328	0.294	0.320	0.253	0.259

Continued on next page

**Threshold**	**Specificity**		**Sensitivity**		**Precision**		**F1 Score**	
	Mean	SD	Mean	SD	Mean	SD	Mean	SD
0.6	0.812	0.191	0.274	0.299	0.274	0.293	0.260	0.271
0.61	0.779	0.217	0.365	0.343	0.331	0.308	0.316	0.270
0.62	0.826	0.163	0.332	0.340	0.281	0.261	0.278	0.256
0.63	0.853	0.165	0.363	0.339	0.331	0.280	0.322	0.277
0.64	0.840	0.213	0.151	0.221	0.185	0.286	0.150	0.208
0.65	0.835	0.207	0.234	0.258	0.326	0.377	0.234	0.223
0.66	0.880	0.201	0.283	0.341	0.304	0.354	0.245	0.261
0.67	0.893	0.154	0.243	0.296	0.314	0.356	0.238	0.262
0.68	0.874	0.146	0.289	0.350	0.325	0.381	0.253	0.292
0.69	0.936	0.140	0.159	0.284	0.229	0.344	0.149	0.222
0.7	0.901	0.135	0.201	0.335	0.245	0.354	0.170	0.250
0.71	0.904	0.180	0.045	0.119	0.128	0.324	0.057	0.149
0.72	0.925	0.148	0.065	0.211	0.070	0.233	0.066	0.217
0.73	0.958	0.105	0.094	0.244	0.120	0.312	0.105	0.271
0.74	0.958	0.069	0.105	0.276	0.105	0.276	0.102	0.264
0.75	0.971	0.088	0.095	0.237	0.119	0.295	0.103	0.253
0.76	0.966	0.084	0.087	0.190	0.116	0.253	0.099	0.217
0.77	0.967	0.101	0.088	0.261	0.070	0.205	0.077	0.226
0.78	0.949	0.093	0.098	0.211	0.121	0.257	0.108	0.230
0.79	0.985	0.034	0.105	0.261	0.096	0.225	0.095	0.221
0.8	0.966	0.065	0.105	0.204	0.140	0.272	0.120	0.233
0.81	0.948	0.110	0.114	0.258	0.114	0.243	0.108	0.230
0.82	0.947	0.115	0.076	0.245	0.053	0.170	0.061	0.192
0.83	0.992	0.026	0.068	0.228	0.053	0.170	0.056	0.179
0.84	0.901	0.154	0.139	0.325	0.102	0.230	0.113	0.256
0.85	0.935	0.147	0.111	0.266	0.167	0.373	0.130	0.297
0.86	0.951	0.119	0.050	0.150	0.100	0.300	0.067	0.200
0.87	1.000	0.000	0.048	0.147	0.095	0.294	0.063	0.196
0.88	1.000	0.000	0.078	0.173	0.176	0.381	0.108	0.235
0.89	0.978	0.092	0.097	0.238	0.222	0.416	0.122	0.259
0.9	0.954	0.129	0.000	0.000	0.000	0.000	0.000	0.000

Table S4: Evaluation metrics by frequency and threshold of CNN SSL model for ID 12. The AUC score is 0.642.

**Threshold**	**Specificity**		**Sensitivity**		**Precision**		**F1 Score**	
	Mean	SD	Mean	SD	Mean	SD	Mean	SD
0.1	0.021	0.060	1.000	0.000	0.385	0.198	0.525	0.216
0.11	0.090	0.130	1.000	0.000	0.363	0.145	0.517	0.150
0.12	0.029	0.072	1.000	0.000	0.352	0.145	0.502	0.179
0.13	0.124	0.174	1.000	0.000	0.381	0.138	0.537	0.145
0.14	0.155	0.205	1.000	0.000	0.409	0.201	0.552	0.204
0.15	0.103	0.158	1.000	0.000	0.379	0.169	0.529	0.171
0.16	0.139	0.195	1.000	0.000	0.443	0.205	0.585	0.203
0.17	0.173	0.226	1.000	0.000	0.414	0.161	0.567	0.162
0.18	0.128	0.209	1.000	0.000	0.363	0.188	0.504	0.212
0.19	0.106	0.188	1.000	0.000	0.395	0.177	0.542	0.197
0.2	0.029	0.100	1.000	0.000	0.380	0.183	0.525	0.198
0.21	0.173	0.231	1.000	0.000	0.470	0.193	0.615	0.187
0.22	0.144	0.225	1.000	0.000	0.415	0.174	0.565	0.180
0.23	0.124	0.233	1.000	0.000	0.358	0.132	0.514	0.135
0.24	0.286	0.252	0.979	0.063	0.417	0.231	0.546	0.244
0.25	0.281	0.234	0.977	0.057	0.458	0.217	0.593	0.199
0.26	0.240	0.247	0.989	0.046	0.444	0.127	0.601	0.129
0.27	0.176	0.237	0.994	0.025	0.426	0.170	0.575	0.170
0.28	0.224	0.259	0.977	0.057	0.427	0.215	0.559	0.218
0.29	0.318	0.328	0.993	0.026	0.475	0.186	0.620	0.173
0.3	0.222	0.266	0.976	0.066	0.420	0.199	0.560	0.200
0.31	0.196	0.266	0.978	0.063	0.418	0.173	0.565	0.161
0.32	0.217	0.265	0.876	0.213	0.349	0.178	0.481	0.189
0.33	0.183	0.279	0.906	0.173	0.387	0.229	0.509	0.234
0.34	0.262	0.289	0.846	0.309	0.394	0.243	0.515	0.259
0.35	0.287	0.292	0.890	0.185	0.477	0.183	0.607	0.171
0.36	0.287	0.287	0.773	0.350	0.434	0.243	0.544	0.269
0.37	0.434	0.337	0.867	0.307	0.507	0.290	0.612	0.281
0.38	0.245	0.232	0.642	0.413	0.347	0.269	0.437	0.310
0.39	0.299	0.354	0.868	0.276	0.394	0.218	0.514	0.226
0.4	0.564	0.377	0.802	0.287	0.513	0.305	0.585	0.294
0.41	0.262	0.266	0.800	0.229	0.427	0.182	0.540	0.175
0.42	0.313	0.348	0.665	0.365	0.345	0.276	0.435	0.287
0.43	0.449	0.379	0.797	0.319	0.510	0.296	0.593	0.279
0.44	0.288	0.344	0.671	0.423	0.355	0.299	0.442	0.321
0.45	0.500	0.342	0.736	0.356	0.529	0.307	0.586	0.302
0.46	0.409	0.348	0.826	0.270	0.478	0.295	0.564	0.273
0.47	0.585	0.292	0.701	0.372	0.511	0.311	0.572	0.314
0.48	0.558	0.314	0.471	0.397	0.308	0.279	0.356	0.305
0.49	0.628	0.312	0.675	0.380	0.500	0.364	0.541	0.354
0.5	0.668	0.252	0.536	0.347	0.429	0.328	0.441	0.302
0.51	0.673	0.286	0.528	0.360	0.380	0.305	0.393	0.259
0.52	0.695	0.281	0.372	0.286	0.419	0.315	0.313	0.192
0.53	0.676	0.241	0.500	0.276	0.437	0.279	0.420	0.211
0.54	0.776	0.232	0.347	0.310	0.330	0.315	0.321	0.286
0.55	0.863	0.193	0.134	0.188	0.189	0.275	0.151	0.211
0.56	0.867	0.164	0.172	0.182	0.280	0.327	0.196	0.201
0.57	0.900	0.174	0.137	0.225	0.262	0.400	0.149	0.203
0.58	0.898	0.150	0.101	0.131	0.266	0.404	0.134	0.182
0.59	0.812	0.194	0.181	0.252	0.245	0.361	0.148	0.173
0.6	0.937	0.126	0.022	0.064	0.069	0.233	0.028	0.079
0.61	0.952	0.099	0.057	0.129	0.058	0.133	0.052	0.112
0.62	0.933	0.119	0.000	0.000	0.000	0.000	0.000	0.000

Continued on next page

Threshold	Specificity		Sensitivity		Precision		F1 Score	
	Mean	SD	Mean	SD	Mean	SD	Mean	SD
0.63	0.956	0.090	0.050	0.100	0.093	0.222	0.062	0.125
0.64	0.967	0.085	0.000	0.000	0.000	0.000	0.000	0.000
0.65	0.986	0.035	0.036	0.087	0.071	0.175	0.048	0.117
0.66	0.949	0.105	0.039	0.091	0.079	0.182	0.053	0.122
0.67	0.990	0.030	0.025	0.075	0.050	0.150	0.033	0.100
0.68	0.942	0.105	0.075	0.225	0.075	0.179	0.067	0.170
0.69	0.983	0.038	0.033	0.084	0.065	0.168	0.043	0.112
0.7	1.000	0.000	0.000	0.000	0.000	0.000	0.000	0.000
0.71	0.986	0.054	0.000	0.000	0.000	0.000	0.000	0.000
0.72	1.000	0.000	0.000	0.000	0.000	0.000	0.000	0.000
0.73	0.995	0.020	0.000	0.000	0.000	0.000	0.000	0.000
0.74	0.973	0.060	0.000	0.000	0.000	0.000	0.000	0.000
0.75	0.987	0.041	0.000	0.000	0.000	0.000	0.000	0.000
0.76	0.989	0.033	0.000	0.000	0.000	0.000	0.000	0.000
0.77	0.995	0.024	0.000	0.000	0.000	0.000	0.000	0.000
0.78	0.976	0.052	0.000	0.000	0.000	0.000	0.000	0.000
0.79	1.000	0.000	0.000	0.000	0.000	0.000	0.000	0.000
0.8	1.000	0.000	0.000	0.000	0.000	0.000	0.000	0.000
0.81	1.000	0.000	0.000	0.000	0.000	0.000	0.000	0.000
0.82	1.000	0.000	0.000	0.000	0.000	0.000	0.000	0.000
0.83	0.993	0.031	0.000	0.000	0.000	0.000	0.000	0.000
0.84	1.000	0.000	0.000	0.000	0.000	0.000	0.000	0.000
0.85	1.000	0.000	0.000	0.000	0.000	0.000	0.000	0.000
0.86	1.000	0.000	0.000	0.000	0.000	0.000	0.000	0.000
0.87	0.997	0.016	0.000	0.000	0.000	0.000	0.000	0.000
0.88	1.000	0.000	0.000	0.000	0.000	0.000	0.000	0.000
0.89	0.993	0.032	0.000	0.000	0.000	0.000	0.000	0.000
0.9	1.000	0.000	0.000	0.000	0.000	0.000	0.000	0.000

ID 13

Table S5: Evaluation metrics by frequency and threshold of CNN control model for ID 13. The AUC score is 0.703.

Threshold	Specificity		Sensitivity		Precision		F1 Score	
	Mean	SD	Mean	SD	Mean	SD	Mean	SD
0.1	0.055	0.112	1.000	0.000	0.322	0.120	0.473	0.146
0.11	0.069	0.122	1.000	0.000	0.244	0.115	0.379	0.150
0.12	0.060	0.101	1.000	0.000	0.211	0.087	0.340	0.122
0.13	0.142	0.179	1.000	0.000	0.245	0.129	0.377	0.161
0.14	0.131	0.156	1.000	0.000	0.281	0.121	0.424	0.150
0.15	0.169	0.189	1.000	0.000	0.265	0.098	0.409	0.126
0.16	0.121	0.152	1.000	0.000	0.231	0.078	0.368	0.108
0.17	0.167	0.169	1.000	0.000	0.221	0.125	0.345	0.165
0.18	0.173	0.212	1.000	0.000	0.258	0.136	0.391	0.174
0.19	0.164	0.196	1.000	0.000	0.233	0.138	0.359	0.177
0.2	0.171	0.223	0.978	0.070	0.234	0.122	0.359	0.152
0.21	0.230	0.221	0.975	0.075	0.293	0.143	0.429	0.164
0.22	0.200	0.238	1.000	0.000	0.265	0.147	0.398	0.182
0.23	0.287	0.262	0.950	0.150	0.272	0.150	0.393	0.181
0.24	0.330	0.259	0.960	0.125	0.294	0.126	0.430	0.149
0.25	0.436	0.258	0.887	0.260	0.363	0.148	0.466	0.160
0.26	0.296	0.298	0.952	0.147	0.235	0.108	0.359	0.142
0.27	0.331	0.324	0.913	0.230	0.323	0.141	0.434	0.154
0.28	0.435	0.326	0.817	0.318	0.352	0.133	0.426	0.137
0.29	0.340	0.242	0.968	0.142	0.308	0.164	0.434	0.181
0.3	0.272	0.324	0.941	0.196	0.294	0.129	0.414	0.147
0.31	0.334	0.336	0.961	0.167	0.376	0.236	0.489	0.237
0.32	0.413	0.288	0.890	0.254	0.292	0.157	0.383	0.166
0.33	0.539	0.289	0.913	0.235	0.383	0.223	0.480	0.227
0.34	0.479	0.234	0.964	0.160	0.379	0.190	0.507	0.203
0.35	0.531	0.322	0.862	0.279	0.506	0.301	0.512	0.199
0.36	0.553	0.272	0.818	0.386	0.283	0.191	0.405	0.249
0.37	0.556	0.322	0.712	0.439	0.226	0.189	0.326	0.246
0.38	0.591	0.324	0.792	0.406	0.325	0.197	0.453	0.255
0.39	0.577	0.282	0.866	0.292	0.369	0.246	0.469	0.236
0.4	0.612	0.283	0.750	0.375	0.284	0.265	0.355	0.246
0.41	0.669	0.294	0.780	0.357	0.403	0.306	0.468	0.268
0.42	0.677	0.315	0.665	0.411	0.319	0.254	0.396	0.258
0.43	0.655	0.274	0.647	0.435	0.315	0.288	0.381	0.299
0.44	0.711	0.236	0.808	0.254	0.460	0.270	0.539	0.228
0.45	0.715	0.252	0.623	0.372	0.391	0.324	0.438	0.308
0.46	0.761	0.273	0.611	0.382	0.466	0.375	0.513	0.373
0.47	0.696	0.276	0.558	0.371	0.374	0.242	0.418	0.267
0.48	0.764	0.235	0.526	0.374	0.433	0.331	0.420	0.286
0.49	0.745	0.239	0.587	0.388	0.388	0.355	0.409	0.320
0.5	0.772	0.215	0.527	0.409	0.354	0.355	0.386	0.341
0.51	0.770	0.184	0.444	0.389	0.333	0.315	0.369	0.336
0.52	0.678	0.217	0.318	0.376	0.139	0.179	0.174	0.203
0.53	0.781	0.201	0.345	0.355	0.275	0.341	0.283	0.308
0.54	0.759	0.260	0.263	0.346	0.173	0.263	0.185	0.259
0.55	0.725	0.210	0.337	0.359	0.199	0.243	0.229	0.259
0.56	0.821	0.165	0.325	0.323	0.251	0.270	0.257	0.244
0.57	0.869	0.201	0.327	0.357	0.296	0.396	0.270	0.322
0.58	0.837	0.214	0.163	0.280	0.101	0.181	0.113	0.192
0.59	0.786	0.223	0.206	0.321	0.182	0.310	0.164	0.260

Continued on next page

**Threshold**	**Specificity**		**Sensitivity**		**Precision**		**F1 Score**	
	Mean	SD	Mean	SD	Mean	SD	Mean	SD
0.6	0.832	0.185	0.229	0.294	0.232	0.331	0.222	0.291
0.61	0.805	0.170	0.266	0.334	0.148	0.182	0.179	0.211
0.62	0.842	0.133	0.116	0.288	0.052	0.116	0.065	0.145
0.63	0.857	0.125	0.045	0.208	0.009	0.042	0.015	0.069
0.64	0.866	0.140	0.139	0.314	0.060	0.114	0.078	0.154
0.65	0.910	0.122	0.058	0.212	0.022	0.070	0.030	0.098
0.66	0.862	0.135	0.054	0.208	0.022	0.070	0.028	0.094
0.67	0.865	0.139	0.062	0.108	0.062	0.108	0.062	0.108
0.68	0.909	0.109	0.107	0.295	0.048	0.125	0.063	0.167
0.69	0.964	0.080	0.000	0.000	0.000	0.000	0.000	0.000
0.7	0.944	0.090	0.000	0.000	0.000	0.000	0.000	0.000
0.71	0.953	0.104	0.000	0.000	0.000	0.000	0.000	0.000
0.72	0.996	0.017	0.000	0.000	0.000	0.000	0.000	0.000
0.73	0.986	0.068	0.000	0.000	0.000	0.000	0.000	0.000
0.74	0.930	0.129	0.000	0.000	0.000	0.000	0.000	0.000
0.75	0.961	0.090	0.000	0.000	0.000	0.000	0.000	0.000
0.76	0.964	0.095	0.000	0.000	0.000	0.000	0.000	0.000
0.77	0.962	0.090	0.000	0.000	0.000	0.000	0.000	0.000
0.78	0.990	0.044	0.000	0.000	0.000	0.000	0.000	0.000
0.79	0.976	0.068	0.000	0.000	0.000	0.000	0.000	0.000
0.8	0.963	0.088	0.000	0.000	0.000	0.000	0.000	0.000
0.81	0.986	0.052	0.000	0.000	0.000	0.000	0.000	0.000
0.82	0.984	0.071	0.000	0.000	0.000	0.000	0.000	0.000
0.83	0.979	0.063	0.000	0.000	0.000	0.000	0.000	0.000
0.84	0.969	0.072	0.000	0.000	0.000	0.000	0.000	0.000
0.85	0.996	0.019	0.000	0.000	0.000	0.000	0.000	0.000
0.86	1.000	0.000	0.000	0.000	0.000	0.000	0.000	0.000
0.87	1.000	0.000	0.000	0.000	0.000	0.000	0.000	0.000
0.88	1.000	0.000	0.000	0.000	0.000	0.000	0.000	0.000
0.89	1.000	0.000	0.000	0.000	0.000	0.000	0.000	0.000
0.9	1.000	0.000	0.000	0.000	0.000	0.000	0.000	0.000

Table S6: Evaluation metrics by frequency and threshold of CNN SSL model for ID 13. The AUC score is 0.714.

Threshold	Specificity		Sensitivity		Precision		F1 Score	
	Mean	SD	Mean	SD	Mean	SD	Mean	SD
0.1	0.104	0.167	1.000	0.000	0.212	0.106	0.338	0.142
0.11	0.164	0.191	1.000	0.000	0.240	0.123	0.371	0.157
0.12	0.223	0.213	1.000	0.000	0.311	0.119	0.461	0.143
0.13	0.140	0.198	1.000	0.000	0.226	0.116	0.353	0.158
0.14	0.290	0.246	1.000	0.000	0.315	0.153	0.458	0.176
0.15	0.276	0.200	1.000	0.000	0.285	0.129	0.429	0.152
0.16	0.240	0.246	1.000	0.000	0.294	0.139	0.437	0.167
0.17	0.285	0.190	1.000	0.000	0.325	0.112	0.479	0.133
0.18	0.510	0.256	1.000	0.000	0.395	0.162	0.545	0.180
0.19	0.429	0.337	1.000	0.000	0.392	0.208	0.531	0.223
0.2	0.343	0.236	1.000	0.000	0.340	0.133	0.491	0.159
0.21	0.339	0.198	1.000	0.000	0.288	0.152	0.425	0.191
0.22	0.347	0.230	1.000	0.000	0.330	0.129	0.481	0.155
0.23	0.432	0.259	1.000	0.000	0.331	0.177	0.471	0.206
0.24	0.500	0.253	1.000	0.000	0.428	0.156	0.581	0.169
0.25	0.469	0.304	1.000	0.000	0.412	0.235	0.548	0.219
0.26	0.461	0.184	1.000	0.000	0.341	0.168	0.483	0.201
0.27	0.472	0.271	1.000	0.000	0.413	0.217	0.552	0.219
0.28	0.407	0.261	1.000	0.000	0.361	0.223	0.493	0.233
0.29	0.446	0.296	0.963	0.112	0.399	0.272	0.506	0.247
0.3	0.461	0.302	0.883	0.168	0.400	0.268	0.485	0.194
0.31	0.435	0.263	0.933	0.122	0.341	0.165	0.472	0.176
0.32	0.498	0.332	0.870	0.212	0.459	0.315	0.527	0.246
0.33	0.577	0.295	0.920	0.217	0.444	0.238	0.562	0.210
0.34	0.503	0.270	0.815	0.337	0.335	0.194	0.457	0.239
0.35	0.654	0.309	0.804	0.339	0.441	0.329	0.490	0.260
0.36	0.630	0.287	0.742	0.398	0.419	0.327	0.486	0.319
0.37	0.621	0.249	0.801	0.376	0.333	0.255	0.425	0.262
0.38	0.661	0.221	0.725	0.436	0.300	0.251	0.402	0.304
0.39	0.494	0.241	0.882	0.291	0.311	0.219	0.429	0.252
0.4	0.596	0.261	0.802	0.310	0.380	0.243	0.480	0.240
0.41	0.591	0.273	0.681	0.388	0.303	0.277	0.370	0.275
0.42	0.713	0.236	0.590	0.411	0.306	0.227	0.370	0.262
0.43	0.593	0.295	0.667	0.383	0.309	0.293	0.380	0.280
0.44	0.704	0.246	0.452	0.403	0.245	0.267	0.300	0.303
0.45	0.586	0.249	0.649	0.404	0.268	0.249	0.350	0.287
0.46	0.707	0.192	0.716	0.406	0.372	0.313	0.453	0.334
0.47	0.760	0.260	0.514	0.460	0.273	0.345	0.305	0.334
0.48	0.804	0.270	0.302	0.415	0.141	0.226	0.179	0.271
0.49	0.699	0.227	0.635	0.400	0.358	0.320	0.411	0.314
0.5	0.742	0.223	0.530	0.451	0.289	0.304	0.350	0.336
0.51	0.783	0.239	0.366	0.445	0.187	0.260	0.232	0.307
0.52	0.799	0.232	0.183	0.378	0.083	0.215	0.101	0.243
0.53	0.668	0.266	0.434	0.409	0.269	0.331	0.298	0.323
0.54	0.819	0.176	0.482	0.465	0.351	0.356	0.399	0.391
0.55	0.762	0.190	0.479	0.427	0.321	0.321	0.356	0.340
0.56	0.844	0.200	0.458	0.439	0.400	0.407	0.384	0.363
0.57	0.902	0.176	0.425	0.413	0.430	0.420	0.407	0.392
0.58	0.809	0.269	0.369	0.431	0.178	0.256	0.217	0.287
0.59	0.811	0.185	0.450	0.465	0.245	0.297	0.290	0.330
0.6	0.779	0.248	0.299	0.420	0.158	0.270	0.162	0.243
0.61	0.821	0.158	0.404	0.401	0.336	0.345	0.325	0.313
0.62	0.789	0.195	0.357	0.408	0.244	0.298	0.261	0.291

Continued on next page

**Threshold**	**Specificity**		**Sensitivity**		**Precision**		**F1 Score**	
	Mean	SD	Mean	SD	Mean	SD	Mean	SD
0.63	0.829	0.192	0.153	0.333	0.137	0.303	0.138	0.302
0.64	0.828	0.179	0.108	0.225	0.107	0.222	0.101	0.203
0.65	0.930	0.133	0.093	0.216	0.153	0.302	0.097	0.187
0.66	0.919	0.131	0.058	0.124	0.152	0.336	0.068	0.131
0.67	0.882	0.153	0.120	0.243	0.120	0.275	0.114	0.243
0.68	0.848	0.148	0.103	0.144	0.180	0.320	0.110	0.153
0.69	0.884	0.142	0.137	0.233	0.288	0.420	0.163	0.250
0.7	0.912	0.153	0.071	0.134	0.207	0.386	0.097	0.179
0.71	0.926	0.137	0.018	0.060	0.087	0.282	0.030	0.098
0.72	0.882	0.149	0.073	0.243	0.125	0.331	0.080	0.247
0.73	0.944	0.107	0.000	0.000	0.000	0.000	0.000	0.000
0.74	0.906	0.149	0.000	0.000	0.000	0.000	0.000	0.000
0.75	0.934	0.141	0.000	0.000	0.000	0.000	0.000	0.000
0.76	0.924	0.117	0.000	0.000	0.000	0.000	0.000	0.000
0.77	0.940	0.118	0.000	0.000	0.000	0.000	0.000	0.000
0.78	0.937	0.116	0.000	0.000	0.000	0.000	0.000	0.000
0.79	0.917	0.122	0.000	0.000	0.000	0.000	0.000	0.000
0.8	0.967	0.090	0.000	0.000	0.000	0.000	0.000	0.000
0.81	0.987	0.048	0.000	0.000	0.000	0.000	0.000	0.000
0.82	0.974	0.060	0.000	0.000	0.000	0.000	0.000	0.000
0.83	0.968	0.074	0.000	0.000	0.000	0.000	0.000	0.000
0.84	0.975	0.060	0.000	0.000	0.000	0.000	0.000	0.000
0.85	0.982	0.049	0.000	0.000	0.000	0.000	0.000	0.000
0.86	0.986	0.054	0.000	0.000	0.000	0.000	0.000	0.000
0.87	0.959	0.081	0.000	0.000	0.000	0.000	0.000	0.000
0.88	0.968	0.074	0.000	0.000	0.000	0.000	0.000	0.000
0.89	1.000	0.000	0.000	0.000	0.000	0.000	0.000	0.000
0.9	1.000	0.000	0.000	0.000	0.000	0.000	0.000	0.000

ID 14

Table S7: Evaluation metrics by frequency and threshold of CNN control model for ID 14. The AUC score is 0.744.

Threshold	Specificity		Sensitivity		Precision		F1 Score	
	Mean	SD	Mean	SD	Mean	SD	Mean	SD
0.1	0.203	0.135	0.820	0.322	0.169	0.093	0.272	0.135
0.11	0.143	0.144	0.871	0.301	0.161	0.073	0.267	0.110
0.12	0.266	0.175	0.831	0.317	0.175	0.090	0.279	0.126
0.13	0.237	0.177	0.894	0.254	0.180	0.064	0.293	0.092
0.14	0.184	0.139	0.909	0.193	0.180	0.042	0.295	0.060
0.15	0.255	0.187	0.933	0.154	0.205	0.062	0.325	0.075
0.16	0.230	0.133	0.940	0.162	0.215	0.077	0.339	0.100
0.17	0.208	0.155	0.987	0.065	0.208	0.078	0.335	0.103
0.18	0.293	0.141	0.972	0.115	0.210	0.104	0.333	0.136
0.19	0.335	0.188	0.883	0.303	0.202	0.104	0.320	0.136
0.2	0.346	0.185	0.945	0.210	0.215	0.098	0.342	0.135
0.21	0.320	0.170	0.905	0.191	0.231	0.101	0.351	0.111
0.22	0.384	0.123	0.847	0.302	0.189	0.088	0.302	0.132
0.23	0.454	0.126	0.864	0.274	0.226	0.108	0.347	0.138
0.24	0.376	0.149	0.905	0.237	0.208	0.073	0.328	0.106
0.25	0.358	0.193	0.848	0.260	0.253	0.131	0.368	0.144
0.26	0.455	0.172	0.712	0.396	0.190	0.104	0.290	0.153
0.27	0.416	0.149	0.793	0.335	0.182	0.095	0.286	0.138
0.28	0.415	0.139	1.000	0.000	0.219	0.058	0.356	0.083
0.29	0.463	0.223	0.739	0.291	0.277	0.105	0.375	0.106
0.3	0.485	0.163	0.883	0.271	0.259	0.123	0.383	0.157
0.31	0.510	0.161	0.773	0.419	0.242	0.156	0.363	0.222
0.32	0.506	0.156	0.821	0.361	0.253	0.137	0.378	0.189
0.33	0.531	0.122	0.763	0.378	0.254	0.150	0.368	0.204
0.34	0.570	0.176	0.694	0.427	0.224	0.181	0.316	0.223
0.35	0.532	0.142	0.848	0.344	0.260	0.150	0.384	0.200
0.36	0.619	0.113	0.678	0.422	0.286	0.190	0.391	0.245
0.37	0.619	0.132	0.717	0.438	0.235	0.174	0.342	0.232
0.38	0.550	0.169	0.799	0.376	0.233	0.150	0.344	0.200
0.39	0.633	0.132	0.614	0.475	0.200	0.169	0.296	0.241
0.4	0.588	0.174	0.680	0.434	0.225	0.174	0.323	0.231
0.41	0.638	0.151	0.667	0.471	0.215	0.186	0.316	0.250
0.42	0.712	0.153	0.709	0.442	0.314	0.259	0.411	0.301
0.43	0.572	0.178	0.886	0.300	0.291	0.188	0.413	0.208
0.44	0.663	0.168	0.867	0.306	0.377	0.189	0.507	0.222
0.45	0.689	0.146	0.639	0.424	0.278	0.239	0.349	0.247
0.46	0.702	0.127	0.798	0.350	0.335	0.201	0.444	0.221
0.47	0.761	0.151	0.725	0.402	0.366	0.221	0.455	0.261
0.48	0.729	0.107	0.737	0.440	0.309	0.223	0.426	0.280
0.49	0.738	0.116	0.453	0.440	0.206	0.210	0.258	0.245
0.5	0.768	0.091	0.809	0.304	0.374	0.113	0.491	0.161
0.51	0.795	0.102	0.826	0.379	0.423	0.282	0.535	0.306
0.52	0.808	0.079	0.592	0.432	0.343	0.261	0.405	0.297
0.53	0.771	0.101	0.784	0.340	0.377	0.231	0.485	0.249
0.54	0.785	0.118	0.762	0.426	0.319	0.240	0.432	0.287
0.55	0.787	0.106	0.725	0.398	0.369	0.238	0.459	0.262
0.56	0.754	0.096	0.670	0.302	0.352	0.203	0.437	0.212
0.57	0.774	0.114	0.603	0.391	0.315	0.256	0.386	0.277
0.58	0.831	0.137	0.514	0.423	0.332	0.350	0.359	0.321
0.59	0.832	0.125	0.650	0.421	0.357	0.310	0.441	0.334

Continued on next page

Threshold	Specificity		Sensitivity		Precision		F1 Score	
	Mean	SD	Mean	SD	Mean	SD	Mean	SD
0.6	0.833	0.118	0.619	0.415	0.337	0.283	0.399	0.293
0.61	0.876	0.082	0.579	0.407	0.382	0.311	0.439	0.311
0.62	0.850	0.092	0.668	0.365	0.389	0.252	0.474	0.281
0.63	0.861	0.078	0.588	0.405	0.399	0.287	0.454	0.320
0.64	0.853	0.097	0.554	0.403	0.372	0.281	0.420	0.313
0.65	0.883	0.090	0.536	0.420	0.363	0.306	0.407	0.319
0.66	0.897	0.086	0.644	0.387	0.514	0.335	0.547	0.333
0.67	0.933	0.080	0.386	0.453	0.343	0.426	0.356	0.427
0.68	0.908	0.070	0.425	0.401	0.412	0.413	0.403	0.382
0.69	0.870	0.092	0.661	0.320	0.537	0.335	0.547	0.256
0.7	0.923	0.068	0.458	0.401	0.404	0.376	0.409	0.351
0.71	0.952	0.051	0.514	0.453	0.465	0.427	0.475	0.419
0.72	0.950	0.058	0.567	0.389	0.610	0.397	0.547	0.361
0.73	0.951	0.053	0.591	0.391	0.640	0.381	0.574	0.350
0.74	0.959	0.071	0.576	0.423	0.593	0.434	0.554	0.401
0.75	0.953	0.068	0.703	0.392	0.630	0.401	0.633	0.370
0.76	0.974	0.039	0.312	0.434	0.283	0.397	0.291	0.403
0.77	0.963	0.064	0.307	0.451	0.213	0.335	0.241	0.360
0.78	0.986	0.030	0.393	0.488	0.369	0.466	0.379	0.473
0.79	0.963	0.054	0.470	0.460	0.404	0.406	0.429	0.422
0.8	0.978	0.043	0.311	0.412	0.303	0.398	0.303	0.400
0.81	0.975	0.044	0.263	0.409	0.211	0.328	0.223	0.343
0.82	0.982	0.035	0.312	0.428	0.306	0.407	0.305	0.410
0.83	0.991	0.026	0.304	0.443	0.295	0.424	0.292	0.421
0.84	0.993	0.023	0.242	0.388	0.280	0.425	0.252	0.387
0.85	0.981	0.056	0.428	0.444	0.489	0.469	0.447	0.447
0.86	0.975	0.054	0.183	0.357	0.205	0.368	0.190	0.360
0.87	0.993	0.032	0.106	0.237	0.227	0.419	0.135	0.271
0.88	0.996	0.017	0.221	0.225	0.529	0.499	0.306	0.303
0.89	1.000	0.000	0.109	0.193	0.261	0.439	0.151	0.262
0.9	1.000	0.000	0.167	0.267	0.333	0.471	0.214	0.319

Table S8: Evaluation metrics by frequency and threshold of CNN SSL model for ID 14. The AUC score is 0.753.

**Threshold**	**Specificity**		**Sensitivity**		**Precision**		**F1 Score**	
	Mean	SD	Mean	SD	Mean	SD	Mean	SD
0.1	0.343	0.191	0.875	0.258	0.214	0.092	0.333	0.125
0.11	0.279	0.191	0.816	0.333	0.176	0.090	0.280	0.132
0.12	0.402	0.177	0.808	0.351	0.225	0.136	0.336	0.175
0.13	0.393	0.141	0.861	0.262	0.224	0.102	0.341	0.129
0.14	0.361	0.177	0.900	0.195	0.232	0.086	0.355	0.102
0.15	0.510	0.109	0.729	0.372	0.227	0.113	0.338	0.163
0.16	0.368	0.181	0.889	0.201	0.239	0.094	0.362	0.112
0.17	0.330	0.201	0.806	0.295	0.216	0.085	0.333	0.122
0.18	0.371	0.196	0.905	0.255	0.218	0.122	0.337	0.166
0.19	0.418	0.193	0.796	0.351	0.216	0.111	0.332	0.159
0.2	0.360	0.214	0.917	0.234	0.235	0.132	0.356	0.167
0.21	0.355	0.144	0.779	0.331	0.218	0.104	0.332	0.148
0.22	0.432	0.150	0.812	0.282	0.232	0.092	0.346	0.124
0.23	0.397	0.202	0.764	0.347	0.230	0.157	0.327	0.188
0.24	0.420	0.213	0.794	0.386	0.171	0.105	0.274	0.159
0.25	0.497	0.150	0.904	0.241	0.262	0.117	0.394	0.150
0.26	0.528	0.144	0.773	0.326	0.259	0.126	0.372	0.167
0.27	0.575	0.128	0.811	0.308	0.311	0.140	0.435	0.171
0.28	0.512	0.219	0.768	0.354	0.253	0.146	0.370	0.190
0.29	0.481	0.170	0.820	0.316	0.228	0.121	0.342	0.162
0.3	0.498	0.151	0.904	0.190	0.303	0.122	0.437	0.147
0.31	0.534	0.171	0.896	0.248	0.269	0.116	0.399	0.142
0.32	0.521	0.143	0.817	0.337	0.271	0.139	0.396	0.186
0.33	0.558	0.160	0.816	0.275	0.318	0.112	0.448	0.138
0.34	0.440	0.204	0.944	0.229	0.227	0.105	0.358	0.141
0.35	0.537	0.158	0.860	0.269	0.313	0.113	0.447	0.147
0.36	0.565	0.182	0.950	0.150	0.320	0.095	0.469	0.112
0.37	0.618	0.156	0.888	0.216	0.372	0.161	0.507	0.177
0.38	0.578	0.159	0.906	0.219	0.286	0.139	0.410	0.162
0.39	0.579	0.175	0.823	0.254	0.342	0.159	0.457	0.176
0.4	0.593	0.199	0.799	0.290	0.338	0.150	0.451	0.176
0.41	0.605	0.169	0.855	0.267	0.282	0.149	0.406	0.170
0.42	0.675	0.160	0.753	0.398	0.304	0.227	0.402	0.232
0.43	0.692	0.199	0.718	0.385	0.334	0.270	0.412	0.239
0.44	0.683	0.228	0.620	0.433	0.316	0.318	0.365	0.281
0.45	0.660	0.219	0.714	0.385	0.322	0.311	0.372	0.244
0.46	0.726	0.173	0.626	0.408	0.347	0.297	0.404	0.271
0.47	0.710	0.152	0.765	0.354	0.366	0.232	0.457	0.219
0.48	0.762	0.156	0.618	0.416	0.317	0.264	0.381	0.265
0.49	0.769	0.166	0.643	0.389	0.327	0.248	0.408	0.262
0.5	0.693	0.146	0.883	0.205	0.342	0.156	0.467	0.167
0.51	0.711	0.191	0.658	0.281	0.338	0.275	0.385	0.196
0.52	0.726	0.156	0.762	0.333	0.372	0.193	0.476	0.219
0.53	0.747	0.154	0.615	0.276	0.363	0.289	0.405	0.215
0.54	0.740	0.173	0.709	0.262	0.448	0.263	0.502	0.208
0.55	0.912	0.095	0.482	0.387	0.457	0.385	0.443	0.342
0.56	0.727	0.167	0.809	0.309	0.372	0.263	0.470	0.244
0.57	0.797	0.144	0.592	0.374	0.335	0.266	0.407	0.283
0.58	0.743	0.116	0.806	0.339	0.361	0.248	0.477	0.263
0.59	0.747	0.184	0.739	0.309	0.413	0.299	0.453	0.226
0.6	0.851	0.136	0.713	0.396	0.447	0.321	0.520	0.322
0.61	0.840	0.126	0.654	0.373	0.400	0.285	0.454	0.259
0.62	0.805	0.113	0.837	0.229	0.491	0.232	0.579	0.185

Continued on next page

Threshold	Specificity		Sensitivity		Precision		F1 Score	
	Mean	SD	Mean	SD	Mean	SD	Mean	SD
0.63	0.858	0.109	0.594	0.390	0.409	0.308	0.439	0.286
0.64	0.890	0.099	0.617	0.399	0.455	0.302	0.480	0.281
0.65	0.901	0.092	0.636	0.322	0.639	0.338	0.584	0.271
0.66	0.910	0.091	0.559	0.403	0.462	0.353	0.456	0.317
0.67	0.862	0.087	0.775	0.339	0.549	0.243	0.606	0.242
0.68	0.946	0.090	0.422	0.465	0.298	0.360	0.334	0.376
0.69	0.966	0.050	0.379	0.373	0.485	0.414	0.368	0.313
0.7	0.921	0.094	0.569	0.399	0.456	0.340	0.448	0.290
0.71	0.921	0.103	0.601	0.433	0.458	0.366	0.488	0.348
0.72	0.919	0.079	0.594	0.408	0.453	0.308	0.500	0.328
0.73	0.926	0.068	0.565	0.466	0.410	0.354	0.460	0.374
0.74	0.927	0.090	0.625	0.471	0.487	0.398	0.528	0.406
0.75	0.972	0.055	0.515	0.479	0.441	0.432	0.460	0.432
0.76	0.959	0.068	0.429	0.426	0.440	0.459	0.407	0.404
0.77	0.973	0.049	0.542	0.470	0.528	0.455	0.527	0.449
0.78	0.996	0.019	0.496	0.480	0.512	0.491	0.502	0.483
0.79	0.989	0.036	0.457	0.432	0.522	0.480	0.480	0.445
0.8	0.983	0.045	0.381	0.396	0.468	0.465	0.415	0.417
0.81	0.991	0.045	0.313	0.360	0.490	0.492	0.360	0.382
0.82	0.969	0.067	0.395	0.339	0.582	0.442	0.441	0.345
0.83	0.996	0.019	0.206	0.325	0.317	0.454	0.241	0.358
0.84	0.987	0.049	0.257	0.376	0.375	0.484	0.292	0.397
0.85	1.000	0.000	0.250	0.323	0.417	0.493	0.306	0.372
0.86	0.992	0.023	0.132	0.220	0.263	0.440	0.175	0.294
0.87	1.000	0.000	0.167	0.289	0.278	0.448	0.204	0.336
0.88	1.000	0.000	0.083	0.186	0.167	0.373	0.111	0.248
0.89	1.000	0.000	0.167	0.276	0.292	0.455	0.208	0.331
0.9	1.000	0.000	0.115	0.252	0.192	0.394	0.141	0.295

ID 15

Table S9: Evaluation metrics by frequency and threshold of CNN control model for ID 15. The AUC score is 0.662.

Threshold	Specificity		Sensitivity		Precision		F1 Score	
	Mean	SD	Mean	SD	Mean	SD	Mean	SD
0.1	0.402	0.241	0.633	0.436	0.215	0.214	0.299	0.255
0.11	0.456	0.229	0.712	0.389	0.229	0.170	0.326	0.213
0.12	0.480	0.216	0.786	0.327	0.310	0.186	0.416	0.209
0.13	0.491	0.221	0.652	0.377	0.269	0.199	0.362	0.247
0.14	0.428	0.215	0.714	0.356	0.230	0.206	0.319	0.223
0.15	0.484	0.253	0.723	0.379	0.264	0.219	0.352	0.250
0.16	0.437	0.238	0.682	0.444	0.186	0.206	0.259	0.194
0.17	0.550	0.206	0.507	0.444	0.232	0.263	0.293	0.282
0.18	0.467	0.219	0.654	0.433	0.238	0.223	0.319	0.251
0.19	0.616	0.130	0.625	0.475	0.197	0.187	0.286	0.243
0.2	0.595	0.202	0.563	0.433	0.263	0.249	0.334	0.284
0.21	0.565	0.231	0.714	0.422	0.280	0.266	0.360	0.267
0.22	0.607	0.199	0.615	0.453	0.254	0.247	0.324	0.270
0.23	0.649	0.227	0.539	0.392	0.339	0.293	0.356	0.249
0.24	0.683	0.170	0.437	0.442	0.290	0.339	0.282	0.272
0.25	0.699	0.179	0.531	0.440	0.310	0.328	0.345	0.315
0.26	0.730	0.185	0.476	0.422	0.314	0.341	0.300	0.242
0.27	0.685	0.223	0.688	0.372	0.372	0.306	0.425	0.267
0.28	0.667	0.196	0.723	0.380	0.330	0.260	0.412	0.256
0.29	0.674	0.223	0.551	0.449	0.249	0.272	0.309	0.283
0.3	0.736	0.172	0.537	0.438	0.246	0.230	0.308	0.258
0.31	0.717	0.170	0.523	0.430	0.299	0.299	0.322	0.248
0.32	0.745	0.188	0.583	0.417	0.300	0.263	0.351	0.246
0.33	0.693	0.226	0.527	0.389	0.308	0.276	0.326	0.214
0.34	0.696	0.217	0.584	0.416	0.269	0.251	0.317	0.218
0.35	0.752	0.199	0.438	0.396	0.255	0.237	0.291	0.245
0.36	0.788	0.166	0.472	0.410	0.277	0.261	0.317	0.256
0.37	0.748	0.211	0.604	0.433	0.290	0.286	0.337	0.247
0.38	0.765	0.189	0.518	0.402	0.297	0.265	0.343	0.270
0.39	0.789	0.181	0.457	0.418	0.258	0.267	0.282	0.248
0.4	0.793	0.196	0.431	0.415	0.240	0.228	0.277	0.245
0.41	0.828	0.148	0.513	0.447	0.292	0.273	0.329	0.267
0.42	0.820	0.145	0.604	0.432	0.311	0.241	0.374	0.249
0.43	0.833	0.157	0.463	0.410	0.334	0.284	0.327	0.244
0.44	0.769	0.184	0.624	0.398	0.310	0.189	0.379	0.216
0.45	0.859	0.135	0.465	0.380	0.362	0.274	0.366	0.259
0.46	0.760	0.190	0.616	0.465	0.235	0.220	0.315	0.257
0.47	0.789	0.187	0.486	0.459	0.207	0.206	0.268	0.241
0.48	0.841	0.152	0.431	0.397	0.282	0.307	0.293	0.254
0.49	0.925	0.102	0.297	0.381	0.259	0.353	0.243	0.301
0.5	0.820	0.169	0.428	0.431	0.185	0.211	0.238	0.238
0.51	0.813	0.141	0.559	0.466	0.232	0.210	0.316	0.262
0.52	0.818	0.134	0.454	0.388	0.238	0.218	0.280	0.220
0.53	0.822	0.179	0.375	0.439	0.151	0.219	0.192	0.218
0.54	0.821	0.149	0.477	0.390	0.226	0.185	0.285	0.210
0.55	0.849	0.177	0.367	0.444	0.156	0.226	0.195	0.238
0.56	0.914	0.121	0.243	0.285	0.217	0.307	0.201	0.232
0.57	0.802	0.140	0.562	0.320	0.445	0.323	0.426	0.215
0.58	0.881	0.128	0.352	0.376	0.179	0.193	0.226	0.232
0.59	0.854	0.140	0.283	0.301	0.233	0.266	0.229	0.223

Continued on next page

Threshold	Specificity		Sensitivity		Precision		F1 Score	
	Mean	SD	Mean	SD	Mean	SD	Mean	SD
0.6	0.811	0.161	0.507	0.386	0.327	0.265	0.350	0.230
0.61	0.894	0.103	0.208	0.242	0.144	0.184	0.163	0.193
0.62	0.900	0.116	0.091	0.248	0.048	0.139	0.059	0.161
0.63	0.843	0.186	0.174	0.233	0.072	0.107	0.097	0.134
0.64	0.876	0.114	0.083	0.178	0.083	0.187	0.076	0.167
0.65	0.860	0.130	0.168	0.271	0.094	0.172	0.107	0.170
0.66	0.925	0.086	0.045	0.144	0.057	0.184	0.050	0.159
0.67	0.916	0.111	0.095	0.191	0.103	0.219	0.094	0.195
0.68	0.872	0.109	0.089	0.166	0.137	0.276	0.097	0.182
0.69	0.917	0.106	0.083	0.172	0.087	0.222	0.066	0.131
0.7	0.903	0.111	0.029	0.094	0.058	0.188	0.039	0.125
0.71	0.950	0.080	0.029	0.088	0.100	0.300	0.045	0.136
0.72	0.956	0.086	0.038	0.090	0.154	0.361	0.061	0.143
0.73	0.984	0.054	0.014	0.067	0.042	0.200	0.021	0.100
0.74	0.969	0.075	0.027	0.068	0.150	0.357	0.045	0.112
0.75	0.968	0.077	0.010	0.035	0.083	0.276	0.019	0.061
0.76	0.966	0.076	0.024	0.057	0.160	0.367	0.042	0.098
0.77	0.987	0.037	0.017	0.058	0.087	0.282	0.028	0.094
0.78	0.985	0.037	0.000	0.000	0.000	0.000	0.000	0.000
0.79	0.975	0.056	0.000	0.000	0.000	0.000	0.000	0.000
0.8	0.994	0.031	0.000	0.000	0.000	0.000	0.000	0.000
0.81	0.982	0.047	0.000	0.000	0.000	0.000	0.000	0.000
0.82	0.994	0.030	0.000	0.000	0.000	0.000	0.000	0.000
0.83	0.992	0.034	0.000	0.000	0.000	0.000	0.000	0.000
0.84	0.987	0.043	0.000	0.000	0.000	0.000	0.000	0.000
0.85	0.991	0.033	0.000	0.000	0.000	0.000	0.000	0.000
0.86	0.985	0.045	0.000	0.000	0.000	0.000	0.000	0.000
0.87	0.993	0.032	0.000	0.000	0.000	0.000	0.000	0.000
0.88	0.985	0.046	0.000	0.000	0.000	0.000	0.000	0.000
0.89	1.000	0.000	0.000	0.000	0.000	0.000	0.000	0.000
0.9	1.000	0.000	0.000	0.000	0.000	0.000	0.000	0.000

Table S10: Evaluation metrics by frequency and threshold of CNN SSL model for ID 15. The AUC score is 0.667.

**Threshold**	**Specificity**		**Sensitivity**		**Precision**		**F1 Score**	
	Mean	SD	Mean	SD	Mean	SD	Mean	SD
0.1	0.011	0.038	1.000	0.000	0.226	0.112	0.356	0.136
0.11	0.017	0.048	1.000	0.000	0.217	0.077	0.350	0.102
0.12	0.033	0.080	1.000	0.000	0.169	0.069	0.283	0.098
0.13	0.092	0.137	1.000	0.000	0.230	0.118	0.360	0.146
0.14	0.053	0.101	1.000	0.000	0.221	0.089	0.353	0.114
0.15	0.126	0.117	1.000	0.000	0.197	0.102	0.319	0.125
0.16	0.108	0.115	0.917	0.186	0.188	0.098	0.304	0.131
0.17	0.088	0.117	0.978	0.102	0.204	0.122	0.323	0.150
0.18	0.068	0.100	0.947	0.147	0.172	0.100	0.282	0.131
0.19	0.109	0.140	0.913	0.190	0.200	0.099	0.320	0.132
0.2	0.130	0.149	0.919	0.161	0.185	0.078	0.300	0.106
0.21	0.205	0.129	0.882	0.191	0.235	0.140	0.355	0.164
0.22	0.222	0.122	0.912	0.164	0.231	0.116	0.356	0.137
0.23	0.211	0.157	0.913	0.165	0.231	0.152	0.345	0.174
0.24	0.191	0.177	0.917	0.234	0.176	0.071	0.291	0.107
0.25	0.257	0.177	0.853	0.325	0.176	0.114	0.281	0.148
0.26	0.264	0.179	0.794	0.338	0.185	0.117	0.289	0.154
0.27	0.227	0.184	0.778	0.309	0.234	0.164	0.334	0.183
0.28	0.261	0.193	0.878	0.267	0.249	0.138	0.372	0.166
0.29	0.343	0.244	0.867	0.289	0.279	0.163	0.398	0.178
0.3	0.309	0.241	0.892	0.285	0.263	0.157	0.388	0.189
0.31	0.363	0.262	0.914	0.266	0.255	0.156	0.379	0.194
0.32	0.309	0.237	0.892	0.181	0.245	0.127	0.367	0.141
0.33	0.417	0.234	0.809	0.345	0.278	0.160	0.400	0.202
0.34	0.503	0.249	0.902	0.251	0.285	0.176	0.407	0.194
0.35	0.372	0.294	0.790	0.372	0.246	0.161	0.357	0.197
0.36	0.544	0.164	0.872	0.321	0.291	0.153	0.425	0.197
0.37	0.510	0.266	0.766	0.333	0.292	0.181	0.392	0.202
0.38	0.520	0.288	0.720	0.363	0.288	0.202	0.377	0.220
0.39	0.551	0.247	0.743	0.305	0.342	0.180	0.442	0.196
0.4	0.503	0.320	0.839	0.223	0.344	0.149	0.449	0.138
0.41	0.629	0.278	0.769	0.286	0.390	0.263	0.451	0.199
0.42	0.554	0.262	0.761	0.226	0.390	0.216	0.476	0.198
0.43	0.646	0.272	0.658	0.349	0.362	0.287	0.421	0.253
0.44	0.654	0.260	0.666	0.314	0.369	0.247	0.425	0.210
0.45	0.627	0.270	0.784	0.286	0.381	0.238	0.452	0.190
0.46	0.679	0.220	0.549	0.415	0.311	0.289	0.359	0.287
0.47	0.727	0.197	0.549	0.395	0.340	0.293	0.372	0.258
0.48	0.710	0.123	0.636	0.310	0.383	0.205	0.445	0.184
0.49	0.727	0.148	0.558	0.403	0.298	0.226	0.349	0.230
0.5	0.777	0.122	0.543	0.443	0.296	0.300	0.356	0.310
0.51	0.808	0.155	0.397	0.405	0.229	0.225	0.270	0.259
0.52	0.802	0.126	0.570	0.450	0.271	0.211	0.345	0.260
0.53	0.822	0.133	0.443	0.364	0.248	0.240	0.288	0.217
0.54	0.768	0.178	0.508	0.446	0.214	0.192	0.281	0.235
0.55	0.778	0.118	0.323	0.310	0.159	0.146	0.203	0.175
0.56	0.820	0.140	0.281	0.429	0.142	0.214	0.179	0.269
0.57	0.892	0.118	0.138	0.250	0.162	0.269	0.130	0.201
0.58	0.857	0.113	0.167	0.344	0.075	0.144	0.099	0.193
0.59	0.889	0.120	0.088	0.171	0.135	0.288	0.090	0.168
0.6	0.916	0.094	0.060	0.129	0.102	0.230	0.068	0.136
0.61	0.942	0.097	0.024	0.062	0.048	0.117	0.031	0.078

Continued on next page

Threshold	Specificity		Sensitivity		Precision		F1 Score	
	Mean	SD	Mean	SD	Mean	SD	Mean	SD
0.62	0.948	0.098	0.054	0.129	0.096	0.235	0.060	0.133
0.63	0.939	0.082	0.093	0.169	0.135	0.250	0.089	0.143
0.64	0.956	0.067	0.055	0.128	0.148	0.328	0.077	0.175
0.65	0.960	0.060	0.054	0.123	0.146	0.305	0.075	0.165
0.66	0.963	0.064	0.030	0.085	0.104	0.288	0.047	0.130
0.67	0.962	0.064	0.031	0.102	0.077	0.228	0.044	0.140
0.68	0.974	0.051	0.034	0.082	0.087	0.190	0.048	0.109
0.69	0.987	0.043	0.000	0.000	0.000	0.000	0.000	0.000
0.7	0.959	0.063	0.017	0.044	0.065	0.168	0.027	0.070
0.71	0.968	0.048	0.060	0.104	0.136	0.223	0.082	0.138
0.72	1.000	0.000	0.007	0.033	0.040	0.196	0.011	0.056
0.73	1.000	0.000	0.023	0.059	0.143	0.350	0.040	0.100
0.74	1.000	0.000	0.000	0.000	0.000	0.000	0.000	0.000
0.75	1.000	0.000	0.000	0.000	0.000	0.000	0.000	0.000
0.76	1.000	0.000	0.000	0.000	0.000	0.000	0.000	0.000
0.77	1.000	0.000	0.000	0.000	0.000	0.000	0.000	0.000
0.78	1.000	0.000	0.000	0.000	0.000	0.000	0.000	0.000
0.79	1.000	0.000	0.000	0.000	0.000	0.000	0.000	0.000
0.8	1.000	0.000	0.000	0.000	0.000	0.000	0.000	0.000
0.81	1.000	0.000	0.000	0.000	0.000	0.000	0.000	0.000
0.82	1.000	0.000	0.000	0.000	0.000	0.000	0.000	0.000
0.83	1.000	0.000	0.000	0.000	0.000	0.000	0.000	0.000
0.84	1.000	0.000	0.000	0.000	0.000	0.000	0.000	0.000
0.85	1.000	0.000	0.000	0.000	0.000	0.000	0.000	0.000
0.86	1.000	0.000	0.000	0.000	0.000	0.000	0.000	0.000
0.87	1.000	0.000	0.000	0.000	0.000	0.000	0.000	0.000
0.88	1.000	0.000	0.000	0.000	0.000	0.000	0.000	0.000
0.89	1.000	0.000	0.000	0.000	0.000	0.000	0.000	0.000
0.9	1.000	0.000	0.000	0.000	0.000	0.000	0.000	0.000

ID 18

Table S11: Evaluation metrics by frequency and threshold of CNN control model for ID 18. The AUC score is 0.808.

Threshold	Specificity		Sensitivity		Precision		F1 Score	
	Mean	SD	Mean	SD	Mean	SD	Mean	SD
0.1	0.245	0.249	1.000	0.000	0.169	0.051	0.287	0.068
0.11	0.232	0.237	1.000	0.000	0.166	0.066	0.280	0.093
0.12	0.183	0.202	1.000	0.000	0.153	0.035	0.263	0.052
0.13	0.179	0.212	1.000	0.000	0.157	0.048	0.268	0.068
0.14	0.309	0.187	1.000	0.000	0.184	0.038	0.309	0.053
0.15	0.307	0.217	1.000	0.000	0.183	0.051	0.307	0.068
0.16	0.359	0.273	1.000	0.000	0.203	0.066	0.332	0.087
0.17	0.292	0.212	1.000	0.000	0.174	0.050	0.294	0.071
0.18	0.373	0.192	1.000	0.000	0.186	0.050	0.311	0.070
0.19	0.349	0.190	1.000	0.000	0.181	0.054	0.303	0.078
0.2	0.365	0.188	1.000	0.000	0.202	0.057	0.333	0.073
0.21	0.430	0.211	1.000	0.000	0.215	0.065	0.350	0.083
0.22	0.363	0.136	1.000	0.000	0.197	0.043	0.327	0.060
0.23	0.452	0.228	1.000	0.000	0.216	0.074	0.350	0.097
0.24	0.452	0.189	0.978	0.102	0.229	0.059	0.367	0.078
0.25	0.440	0.164	0.950	0.150	0.204	0.078	0.331	0.103
0.26	0.487	0.182	0.941	0.161	0.199	0.060	0.326	0.086
0.27	0.458	0.167	0.935	0.168	0.205	0.060	0.333	0.086
0.28	0.533	0.170	0.972	0.115	0.248	0.088	0.389	0.108
0.29	0.458	0.193	0.957	0.141	0.223	0.087	0.355	0.113
0.3	0.482	0.147	0.882	0.322	0.194	0.085	0.316	0.132
0.31	0.558	0.213	0.824	0.381	0.227	0.162	0.343	0.205
0.32	0.560	0.137	0.889	0.314	0.219	0.099	0.348	0.146
0.33	0.566	0.157	0.909	0.287	0.231	0.095	0.365	0.138
0.34	0.542	0.185	0.950	0.218	0.241	0.102	0.377	0.135
0.35	0.572	0.171	0.950	0.218	0.259	0.113	0.399	0.144
0.36	0.578	0.176	1.000	0.000	0.283	0.125	0.428	0.134
0.37	0.516	0.171	0.882	0.322	0.218	0.097	0.346	0.145
0.38	0.618	0.187	0.950	0.218	0.281	0.140	0.421	0.164
0.39	0.692	0.152	0.840	0.367	0.274	0.158	0.405	0.208
0.4	0.637	0.139	0.826	0.379	0.237	0.128	0.364	0.187
0.41	0.674	0.156	0.810	0.393	0.274	0.182	0.399	0.229
0.42	0.677	0.185	0.944	0.229	0.336	0.202	0.474	0.199
0.43	0.681	0.124	0.850	0.357	0.278	0.207	0.400	0.226
0.44	0.630	0.140	0.941	0.235	0.270	0.136	0.408	0.165
0.45	0.709	0.168	0.864	0.343	0.276	0.140	0.412	0.192
0.46	0.665	0.113	0.905	0.294	0.272	0.113	0.413	0.157
0.47	0.663	0.135	0.875	0.298	0.299	0.181	0.431	0.191
0.48	0.672	0.134	0.763	0.376	0.245	0.126	0.365	0.180
0.49	0.689	0.141	0.848	0.273	0.311	0.137	0.442	0.161
0.5	0.703	0.131	0.824	0.340	0.275	0.151	0.405	0.192
0.51	0.717	0.156	0.763	0.376	0.295	0.221	0.406	0.238
0.52	0.735	0.121	0.595	0.397	0.213	0.161	0.304	0.211
0.53	0.684	0.119	0.905	0.196	0.297	0.068	0.436	0.072
0.54	0.718	0.174	0.833	0.333	0.354	0.273	0.463	0.267
0.55	0.789	0.100	0.575	0.363	0.267	0.214	0.341	0.203
0.56	0.803	0.129	0.667	0.356	0.308	0.202	0.406	0.223
0.57	0.751	0.173	0.775	0.370	0.316	0.222	0.416	0.211
0.58	0.797	0.137	0.739	0.325	0.369	0.238	0.456	0.206
0.59	0.854	0.139	0.579	0.437	0.292	0.258	0.372	0.291

Continued on next page

**Threshold**	**Specificity**		**Sensitivity**		**Precision**		**F1 Score**	
	Mean	SD	Mean	SD	Mean	SD	Mean	SD
0.6	0.863	0.141	0.583	0.373	0.384	0.298	0.418	0.259
0.61	0.815	0.124	0.708	0.320	0.384	0.238	0.473	0.234
0.62	0.849	0.118	0.591	0.325	0.382	0.264	0.418	0.212
0.63	0.873	0.115	0.550	0.384	0.332	0.248	0.387	0.245
0.64	0.889	0.106	0.250	0.300	0.220	0.320	0.210	0.253
0.65	0.894	0.101	0.354	0.467	0.154	0.200	0.211	0.273
0.66	0.907	0.077	0.211	0.374	0.100	0.177	0.135	0.239
0.67	0.914	0.098	0.333	0.449	0.173	0.259	0.222	0.311
0.68	0.914	0.078	0.333	0.449	0.168	0.226	0.223	0.300
0.69	0.927	0.085	0.053	0.153	0.035	0.102	0.042	0.123
0.7	0.943	0.060	0.083	0.186	0.062	0.143	0.071	0.159
0.71	0.962	0.067	0.094	0.195	0.062	0.130	0.075	0.156
0.72	0.952	0.057	0.104	0.249	0.056	0.124	0.071	0.159
0.73	0.966	0.059	0.136	0.269	0.106	0.233	0.108	0.204
0.74	0.977	0.035	0.174	0.280	0.217	0.356	0.188	0.300
0.75	0.981	0.033	0.083	0.186	0.125	0.298	0.097	0.220
0.76	0.980	0.034	0.204	0.281	0.315	0.434	0.241	0.325
0.77	0.993	0.023	0.119	0.213	0.190	0.361	0.143	0.259
0.78	0.994	0.021	0.062	0.165	0.083	0.236	0.069	0.186
0.79	1.000	0.000	0.036	0.129	0.071	0.258	0.048	0.172
0.8	0.989	0.027	0.071	0.175	0.071	0.175	0.071	0.175
0.81	0.996	0.017	0.025	0.109	0.025	0.109	0.025	0.109
0.82	1.000	0.000	0.075	0.179	0.150	0.357	0.100	0.238
0.83	1.000	0.000	0.024	0.106	0.048	0.213	0.032	0.142
0.84	1.000	0.000	0.000	0.000	0.000	0.000	0.000	0.000
0.85	1.000	0.000	0.000	0.000	0.000	0.000	0.000	0.000
0.86	1.000	0.000	0.000	0.000	0.000	0.000	0.000	0.000
0.87	1.000	0.000	0.000	0.000	0.000	0.000	0.000	0.000
0.88	1.000	0.000	0.000	0.000	0.000	0.000	0.000	0.000
0.89	1.000	0.000	0.000	0.000	0.000	0.000	0.000	0.000
0.9	1.000	0.000	0.000	0.000	0.000	0.000	0.000	0.000

Table S12: Evaluation metrics by frequency and threshold of CNN SSL model for ID 18. The AUC score is 0.834.

Threshold	Specificity		Sensitivity		Precision		F1 Score	
	Mean	SD	Mean	SD	Mean	SD	Mean	SD
0.1	0.185	0.195	1.000	0.000	0.155	0.032	0.268	0.046
0.11	0.232	0.217	1.000	0.000	0.159	0.060	0.270	0.090
0.12	0.317	0.229	1.000	0.000	0.188	0.052	0.314	0.070
0.13	0.251	0.230	1.000	0.000	0.169	0.049	0.286	0.070
0.14	0.276	0.203	1.000	0.000	0.169	0.029	0.288	0.041
0.15	0.288	0.177	1.000	0.000	0.186	0.041	0.312	0.057
0.16	0.386	0.209	1.000	0.000	0.200	0.060	0.329	0.080
0.17	0.373	0.204	1.000	0.000	0.200	0.064	0.330	0.083
0.18	0.455	0.184	1.000	0.000	0.240	0.079	0.381	0.095
0.19	0.342	0.174	1.000	0.000	0.192	0.038	0.320	0.053
0.2	0.440	0.214	1.000	0.000	0.238	0.110	0.374	0.120
0.21	0.497	0.202	1.000	0.000	0.245	0.088	0.386	0.106
0.22	0.455	0.215	1.000	0.000	0.222	0.072	0.358	0.093
0.23	0.563	0.199	1.000	0.000	0.286	0.127	0.432	0.134
0.24	0.569	0.201	1.000	0.000	0.284	0.124	0.429	0.138
0.25	0.530	0.190	1.000	0.000	0.255	0.076	0.401	0.097
0.26	0.543	0.227	1.000	0.000	0.283	0.106	0.431	0.124
0.27	0.628	0.140	1.000	0.000	0.294	0.107	0.445	0.121
0.28	0.527	0.221	1.000	0.000	0.275	0.145	0.414	0.153
0.29	0.521	0.196	1.000	0.000	0.238	0.055	0.382	0.072
0.3	0.628	0.187	1.000	0.000	0.329	0.173	0.474	0.161
0.31	0.572	0.206	1.000	0.000	0.273	0.100	0.420	0.119
0.32	0.656	0.187	1.000	0.000	0.338	0.184	0.482	0.170
0.33	0.593	0.200	1.000	0.000	0.329	0.197	0.469	0.179
0.34	0.551	0.184	1.000	0.000	0.268	0.092	0.415	0.109
0.35	0.582	0.200	1.000	0.000	0.313	0.183	0.455	0.161
0.36	0.558	0.214	1.000	0.000	0.288	0.123	0.434	0.138
0.37	0.603	0.185	1.000	0.000	0.289	0.075	0.443	0.091
0.38	0.663	0.163	0.925	0.179	0.298	0.112	0.440	0.140
0.39	0.648	0.188	0.850	0.278	0.268	0.116	0.394	0.142
0.4	0.715	0.133	0.880	0.214	0.359	0.186	0.488	0.176
0.41	0.652	0.151	0.857	0.274	0.244	0.079	0.375	0.114
0.42	0.640	0.137	0.944	0.157	0.293	0.084	0.434	0.090
0.43	0.638	0.175	0.870	0.220	0.287	0.112	0.415	0.127
0.44	0.672	0.175	0.789	0.295	0.274	0.108	0.391	0.129
0.45	0.664	0.157	0.875	0.217	0.324	0.180	0.429	0.093
0.46	0.711	0.137	0.833	0.276	0.282	0.099	0.408	0.115
0.47	0.746	0.167	0.771	0.249	0.356	0.137	0.451	0.118
0.48	0.688	0.123	0.750	0.296	0.286	0.104	0.402	0.128
0.49	0.662	0.142	0.568	0.460	0.216	0.187	0.303	0.247
0.5	0.727	0.140	0.632	0.392	0.248	0.156	0.340	0.195
0.51	0.717	0.152	0.500	0.474	0.151	0.144	0.230	0.218
0.52	0.708	0.162	0.625	0.415	0.236	0.159	0.326	0.200
0.53	0.757	0.193	0.413	0.481	0.140	0.180	0.205	0.249
0.54	0.734	0.150	0.500	0.477	0.187	0.183	0.265	0.252
0.55	0.802	0.152	0.559	0.450	0.232	0.244	0.312	0.276
0.56	0.790	0.175	0.364	0.431	0.165	0.200	0.220	0.258
0.57	0.760	0.158	0.479	0.489	0.177	0.183	0.257	0.264
0.58	0.777	0.147	0.587	0.481	0.237	0.208	0.333	0.282
0.59	0.786	0.110	0.700	0.440	0.293	0.200	0.409	0.266
0.6	0.855	0.149	0.447	0.484	0.193	0.219	0.266	0.294
0.61	0.838	0.162	0.333	0.408	0.198	0.279	0.243	0.316

Continued on next page

**Threshold**	**Specificity**		**Sensitivity**		**Precision**		**F1 Score**	
	Mean	SD	Mean	SD	Mean	SD	Mean	SD
0.62	0.879	0.110	0.526	0.472	0.274	0.275	0.353	0.329
0.63	0.882	0.095	0.525	0.487	0.247	0.244	0.331	0.316
0.64	0.853	0.098	0.548	0.460	0.278	0.260	0.361	0.312
0.65	0.870	0.077	0.875	0.311	0.476	0.224	0.606	0.244
0.66	0.915	0.071	0.500	0.500	0.269	0.279	0.346	0.352
0.67	0.957	0.050	0.457	0.487	0.290	0.312	0.354	0.378
0.68	0.944	0.058	0.433	0.442	0.278	0.277	0.331	0.330
0.69	0.923	0.092	0.306	0.378	0.296	0.379	0.274	0.319
0.7	0.932	0.085	0.350	0.391	0.333	0.408	0.322	0.368
0.71	0.916	0.092	0.237	0.250	0.289	0.362	0.247	0.272
0.72	0.981	0.048	0.150	0.229	0.275	0.432	0.192	0.295
0.73	0.969	0.060	0.023	0.104	0.023	0.104	0.023	0.104
0.74	0.969	0.055	0.068	0.172	0.068	0.172	0.068	0.172
0.75	0.967	0.051	0.119	0.213	0.119	0.213	0.119	0.213
0.76	0.988	0.036	0.040	0.136	0.040	0.136	0.040	0.136
0.77	0.993	0.023	0.000	0.000	0.000	0.000	0.000	0.000
0.78	1.000	0.000	0.000	0.000	0.000	0.000	0.000	0.000
0.79	1.000	0.000	0.000	0.000	0.000	0.000	0.000	0.000
0.8	1.000	0.000	0.000	0.000	0.000	0.000	0.000	0.000
0.81	1.000	0.000	0.000	0.000	0.000	0.000	0.000	0.000
0.82	1.000	0.000	0.000	0.000	0.000	0.000	0.000	0.000
0.83	1.000	0.000	0.000	0.000	0.000	0.000	0.000	0.000
0.84	1.000	0.000	0.000	0.000	0.000	0.000	0.000	0.000
0.85	1.000	0.000	0.000	0.000	0.000	0.000	0.000	0.000
0.86	1.000	0.000	0.000	0.000	0.000	0.000	0.000	0.000
0.87	1.000	0.000	0.000	0.000	0.000	0.000	0.000	0.000
0.88	1.000	0.000	0.000	0.000	0.000	0.000	0.000	0.000
0.89	1.000	0.000	0.000	0.000	0.000	0.000	0.000	0.000
0.9	1.000	0.000	0.000	0.000	0.000	0.000	0.000	0.000

ID 20

Table S13: Evaluation metrics by frequency and threshold of CNN control model for ID 20. The AUC score is 0.879.

Threshold	Specificity		Sensitivity		Precision		F1 Score	
	Mean	SD	Mean	SD	Mean	SD	Mean	SD
0.1	0.000	0.000	1.000	0.000	0.698	0.197	0.804	0.159
0.11	0.000	0.000	1.000	0.000	0.614	0.242	0.729	0.213
0.12	0.064	0.138	1.000	0.000	0.625	0.214	0.745	0.188
0.13	0.069	0.155	1.000	0.000	0.605	0.251	0.718	0.229
0.14	0.069	0.148	1.000	0.000	0.614	0.266	0.721	0.242
0.15	0.053	0.128	1.000	0.000	0.698	0.186	0.805	0.150
0.16	0.036	0.129	1.000	0.000	0.664	0.217	0.774	0.187
0.17	0.087	0.163	1.000	0.000	0.611	0.220	0.732	0.192
0.18	0.155	0.259	1.000	0.000	0.717	0.200	0.814	0.182
0.19	0.033	0.160	1.000	0.000	0.688	0.227	0.788	0.199
0.2	0.042	0.161	1.000	0.000	0.651	0.205	0.767	0.180
0.21	0.059	0.235	1.000	0.000	0.784	0.111	0.875	0.074
0.22	0.292	0.455	1.000	0.000	0.715	0.302	0.789	0.260
0.23	0.176	0.381	1.000	0.000	0.735	0.229	0.823	0.187
0.24	0.286	0.452	1.000	0.000	0.722	0.269	0.806	0.214
0.25	0.222	0.416	1.000	0.000	0.787	0.221	0.858	0.183
0.26	0.143	0.350	1.000	0.000	0.738	0.189	0.834	0.137
0.27	0.348	0.476	1.000	0.000	0.819	0.190	0.887	0.134
0.28	0.316	0.465	1.000	0.000	0.737	0.272	0.814	0.225
0.29	0.485	0.457	1.000	0.000	0.812	0.240	0.871	0.189
0.3	0.295	0.397	1.000	0.000	0.675	0.265	0.773	0.215
0.31	0.360	0.376	1.000	0.000	0.746	0.172	0.843	0.118
0.32	0.373	0.418	1.000	0.000	0.734	0.247	0.819	0.194
0.33	0.285	0.358	1.000	0.000	0.698	0.208	0.803	0.159
0.34	0.322	0.436	1.000	0.000	0.809	0.178	0.882	0.133
0.35	0.358	0.409	1.000	0.000	0.793	0.185	0.871	0.140
0.36	0.448	0.447	1.000	0.000	0.796	0.232	0.863	0.181
0.37	0.578	0.456	1.000	0.000	0.850	0.205	0.902	0.152
0.38	0.306	0.426	0.927	0.096	0.819	0.154	0.857	0.123
0.39	0.418	0.468	0.926	0.096	0.835	0.168	0.867	0.128
0.4	0.583	0.470	0.920	0.098	0.887	0.140	0.894	0.091
0.41	0.429	0.495	0.900	0.100	0.879	0.111	0.886	0.092
0.42	0.537	0.473	0.933	0.094	0.880	0.135	0.901	0.104
0.43	0.460	0.477	0.746	0.320	0.729	0.319	0.728	0.305
0.44	0.487	0.482	0.826	0.220	0.842	0.207	0.821	0.192
0.45	0.600	0.455	0.740	0.307	0.775	0.297	0.730	0.265
0.46	0.562	0.496	0.744	0.252	0.852	0.239	0.780	0.228
0.47	0.578	0.463	0.867	0.133	0.871	0.151	0.853	0.094
0.48	0.636	0.457	0.725	0.330	0.786	0.325	0.741	0.308
0.49	0.783	0.398	0.635	0.362	0.746	0.384	0.673	0.354
0.5	0.478	0.500	0.649	0.216	0.827	0.218	0.717	0.205
0.51	0.700	0.458	0.439	0.313	0.774	0.345	0.520	0.295
0.52	0.567	0.473	0.597	0.288	0.816	0.232	0.656	0.242
0.53	0.544	0.465	0.573	0.349	0.662	0.350	0.603	0.333
0.54	0.538	0.466	0.590	0.281	0.804	0.224	0.650	0.230
0.55	0.696	0.427	0.685	0.330	0.765	0.323	0.712	0.313
0.56	0.863	0.325	0.632	0.322	0.844	0.319	0.697	0.295
0.57	0.842	0.365	0.524	0.389	0.653	0.449	0.570	0.401
0.58	1.000	0.000	0.510	0.412	0.650	0.477	0.561	0.427
0.59	1.000	0.000	0.426	0.306	0.706	0.456	0.523	0.354

Continued on next page

Threshold	Specificity		Sensitivity		Precision		F1 Score	
	Mean	SD	Mean	SD	Mean	SD	Mean	SD
0.6	1.000	0.000	0.675	0.263	0.938	0.242	0.769	0.241
0.61	1.000	0.000	0.533	0.411	0.667	0.471	0.583	0.426
0.62	1.000	0.000	0.533	0.227	0.933	0.249	0.664	0.223
0.63	1.000	0.000	0.410	0.397	0.600	0.490	0.468	0.411
0.64	1.000	0.000	0.450	0.415	0.625	0.484	0.502	0.421
0.65	1.000	0.000	0.611	0.352	0.842	0.365	0.685	0.340
0.66	1.000	0.000	0.557	0.396	0.714	0.452	0.614	0.407
0.67	1.000	0.000	0.595	0.398	0.750	0.433	0.648	0.400
0.68	1.000	0.000	0.481	0.375	0.762	0.426	0.556	0.376
0.69	1.000	0.000	0.377	0.356	0.636	0.481	0.451	0.382
0.7	1.000	0.000	0.405	0.355	0.700	0.458	0.484	0.373
0.71	1.000	0.000	0.478	0.385	0.700	0.458	0.545	0.396
0.72	1.000	0.000	0.253	0.217	0.647	0.478	0.354	0.287
0.73	1.000	0.000	0.318	0.224	0.773	0.419	0.436	0.278
0.74	1.000	0.000	0.248	0.192	0.688	0.464	0.357	0.263
0.75	1.000	0.000	0.139	0.213	0.333	0.471	0.191	0.283
0.76	1.000	0.000	0.193	0.202	0.556	0.497	0.277	0.272
0.77	1.000	0.000	0.175	0.248	0.368	0.482	0.232	0.317
0.78	1.000	0.000	0.085	0.171	0.200	0.400	0.119	0.239
0.79	1.000	0.000	0.248	0.236	0.562	0.496	0.339	0.312
0.8	1.000	0.000	0.260	0.238	0.562	0.496	0.354	0.317
0.81	1.000	0.000	0.100	0.158	0.312	0.464	0.149	0.230
0.82	1.000	0.000	0.150	0.206	0.364	0.481	0.210	0.285
0.83	1.000	0.000	0.114	0.127	0.476	0.499	0.183	0.199
0.84	1.000	0.000	0.033	0.087	0.136	0.343	0.053	0.137
0.85	1.000	0.000	0.000	0.000	0.000	0.000	0.000	0.000
0.86	1.000	0.000	0.000	0.000	0.000	0.000	0.000	0.000
0.87	1.000	0.000	0.000	0.000	0.000	0.000	0.000	0.000
0.88	1.000	0.000	0.000	0.000	0.000	0.000	0.000	0.000
0.89	1.000	0.000	0.000	0.000	0.000	0.000	0.000	0.000
0.9	1.000	0.000	0.000	0.000	0.000	0.000	0.000	0.000

Table S14: Evaluation metrics by frequency and threshold of CNN SSL model for ID 20. The AUC score is 0.980.

**Threshold**	**Specificity**		**Sensitivity**		**Precision**		**F1 Score**	
	Mean	SD	Mean	SD	Mean	SD	Mean	SD
0.1	0.085	0.143	1.000	0.000	0.649	0.257	0.751	0.229
0.11	0.128	0.164	1.000	0.000	0.654	0.208	0.769	0.173
0.12	0.169	0.185	1.000	0.000	0.678	0.197	0.788	0.170
0.13	0.208	0.206	1.000	0.000	0.659	0.212	0.771	0.182
0.14	0.189	0.258	1.000	0.000	0.745	0.130	0.847	0.094
0.15	0.368	0.377	1.000	0.000	0.747	0.142	0.847	0.095
0.16	0.176	0.289	1.000	0.000	0.744	0.123	0.847	0.087
0.17	0.231	0.299	1.000	0.000	0.760	0.110	0.859	0.073
0.18	0.402	0.424	1.000	0.000	0.831	0.122	0.902	0.080
0.19	0.501	0.391	1.000	0.000	0.792	0.140	0.877	0.093
0.2	0.330	0.414	1.000	0.000	0.812	0.126	0.891	0.085
0.21	0.461	0.413	1.000	0.000	0.839	0.104	0.909	0.061
0.22	0.618	0.365	1.000	0.000	0.754	0.188	0.846	0.128
0.23	0.570	0.387	1.000	0.000	0.820	0.147	0.894	0.096
0.24	0.666	0.352	1.000	0.000	0.795	0.176	0.874	0.117
0.25	0.641	0.351	1.000	0.000	0.776	0.166	0.864	0.109
0.26	0.413	0.353	1.000	0.000	0.806	0.078	0.890	0.047
0.27	0.425	0.409	1.000	0.000	0.770	0.168	0.859	0.116
0.28	0.498	0.449	1.000	0.000	0.852	0.131	0.914	0.083
0.29	0.529	0.433	1.000	0.000	0.810	0.157	0.886	0.102
0.3	0.457	0.403	0.931	0.117	0.798	0.126	0.852	0.100
0.31	0.429	0.413	0.929	0.120	0.794	0.139	0.847	0.109
0.32	0.501	0.439	0.945	0.103	0.818	0.156	0.866	0.112
0.33	0.424	0.364	0.945	0.096	0.745	0.126	0.822	0.084
0.34	0.627	0.453	0.940	0.113	0.901	0.114	0.909	0.069
0.35	0.629	0.388	0.961	0.082	0.873	0.115	0.906	0.059
0.36	0.525	0.465	0.916	0.129	0.876	0.127	0.882	0.086
0.37	0.626	0.430	0.973	0.083	0.861	0.157	0.901	0.100
0.38	0.503	0.469	0.940	0.123	0.873	0.128	0.891	0.082
0.39	0.568	0.431	0.970	0.087	0.862	0.135	0.903	0.084
0.4	0.713	0.408	0.933	0.128	0.924	0.098	0.918	0.074
0.41	0.669	0.429	0.953	0.102	0.895	0.135	0.912	0.090
0.42	0.576	0.454	0.949	0.110	0.889	0.112	0.907	0.069
0.43	0.486	0.440	0.968	0.079	0.831	0.141	0.883	0.081
0.44	0.555	0.470	0.959	0.087	0.881	0.130	0.909	0.082
0.45	0.560	0.449	0.966	0.080	0.875	0.129	0.909	0.076
0.46	0.523	0.453	0.924	0.116	0.846	0.148	0.869	0.093
0.47	0.444	0.436	0.969	0.089	0.820	0.134	0.878	0.086
0.48	0.697	0.396	0.956	0.083	0.867	0.154	0.897	0.093
0.49	0.732	0.402	0.940	0.103	0.920	0.110	0.923	0.080
0.5	0.456	0.458	0.935	0.111	0.885	0.090	0.901	0.055
0.51	0.642	0.416	0.973	0.068	0.873	0.141	0.912	0.092
0.52	0.540	0.411	0.975	0.081	0.825	0.145	0.883	0.095
0.53	0.571	0.495	0.956	0.095	0.924	0.089	0.936	0.077
0.54	0.750	0.433	0.959	0.094	0.958	0.072	0.954	0.059
0.55	0.688	0.464	0.935	0.114	0.948	0.077	0.934	0.064
0.56	1.000	0.000	0.988	0.048	1.000	0.000	0.993	0.027
0.57	1.000	0.000	0.961	0.086	1.000	0.000	0.978	0.049
0.58	1.000	0.000	0.904	0.144	1.000	0.000	0.943	0.085
0.59	1.000	0.000	0.951	0.101	1.000	0.000	0.972	0.059
0.6	1.000	0.000	0.947	0.112	1.000	0.000	0.969	0.066
0.61	1.000	0.000	0.950	0.090	1.000	0.000	0.972	0.050

Continued on next page

Threshold	Specificity		Sensitivity		Precision		F1 Score	
	Mean	SD	Mean	SD	Mean	SD	Mean	SD
0.62	1.000	0.000	0.910	0.117	1.000	0.000	0.949	0.068
0.63	1.000	0.000	0.946	0.108	1.000	0.000	0.969	0.063
0.64	1.000	0.000	0.989	0.045	1.000	0.000	0.994	0.025
0.65	1.000	0.000	0.965	0.076	1.000	0.000	0.980	0.042
0.66	1.000	0.000	0.947	0.112	1.000	0.000	0.969	0.066
0.67	1.000	0.000	0.900	0.100	1.000	0.000	0.944	0.056
0.68	1.000	0.000	0.843	0.116	1.000	0.000	0.911	0.068
0.69	1.000	0.000	0.863	0.114	1.000	0.000	0.923	0.066
0.7	1.000	0.000	0.903	0.110	1.000	0.000	0.945	0.062
0.71	1.000	0.000	0.875	0.171	1.000	0.000	0.923	0.106
0.72	1.000	0.000	0.890	0.171	1.000	0.000	0.932	0.106
0.73	1.000	0.000	0.805	0.170	1.000	0.000	0.882	0.106
0.74	1.000	0.000	0.819	0.187	1.000	0.000	0.888	0.116
0.75	1.000	0.000	0.882	0.154	1.000	0.000	0.930	0.095
0.76	1.000	0.000	0.819	0.188	1.000	0.000	0.889	0.117
0.77	1.000	0.000	0.812	0.178	1.000	0.000	0.885	0.110
0.78	1.000	0.000	0.854	0.166	1.000	0.000	0.912	0.102
0.79	1.000	0.000	0.786	0.176	1.000	0.000	0.870	0.110
0.8	1.000	0.000	0.756	0.172	1.000	0.000	0.850	0.108
0.81	1.000	0.000	0.730	0.155	1.000	0.000	0.835	0.097
0.82	1.000	0.000	0.681	0.191	1.000	0.000	0.795	0.128
0.83	1.000	0.000	0.818	0.189	1.000	0.000	0.887	0.123
0.84	1.000	0.000	0.692	0.187	1.000	0.000	0.804	0.131
0.85	1.000	0.000	0.630	0.232	1.000	0.000	0.745	0.199
0.86	1.000	0.000	0.604	0.247	0.933	0.249	0.717	0.242
0.87	1.000	0.000	0.512	0.197	1.000	0.000	0.654	0.182
0.88	1.000	0.000	0.568	0.150	1.000	0.000	0.711	0.139
0.89	1.000	0.000	0.556	0.169	0.947	0.223	0.695	0.188
0.9	1.000	0.000	0.597	0.085	1.000	0.000	0.744	0.073

ID 27

Table S15: Evaluation metrics by frequency and threshold of CNN control model for ID 27. The AUC score is 0.638.

Threshold	Specificity		Sensitivity		Precision		F1 Score	
	Mean	SD	Mean	SD	Mean	SD	Mean	SD
0.1	0.010	0.052	1.000	0.000	0.313	0.199	0.442	0.228
0.11	0.012	0.058	1.000	0.000	0.280	0.168	0.412	0.199
0.12	0.053	0.145	1.000	0.000	0.304	0.169	0.442	0.188
0.13	0.000	0.000	0.987	0.047	0.252	0.153	0.377	0.188
0.14	0.025	0.075	0.962	0.111	0.320	0.191	0.453	0.197
0.15	0.022	0.071	0.972	0.099	0.324	0.199	0.454	0.209
0.16	0.113	0.191	0.981	0.048	0.497	0.231	0.626	0.211
0.17	0.165	0.178	0.959	0.085	0.403	0.229	0.533	0.223
0.18	0.122	0.208	0.986	0.040	0.431	0.239	0.559	0.233
0.19	0.200	0.254	0.949	0.152	0.328	0.268	0.435	0.274
0.2	0.169	0.204	0.886	0.201	0.336	0.228	0.442	0.229
0.21	0.208	0.285	0.913	0.241	0.399	0.275	0.515	0.262
0.22	0.146	0.211	0.916	0.229	0.314	0.212	0.436	0.226
0.23	0.194	0.214	0.960	0.097	0.299	0.175	0.423	0.187
0.24	0.187	0.181	0.911	0.242	0.283	0.157	0.411	0.190
0.25	0.321	0.282	0.847	0.300	0.396	0.326	0.477	0.316
0.26	0.295	0.298	0.800	0.324	0.393	0.305	0.474	0.301
0.27	0.209	0.204	0.902	0.201	0.313	0.162	0.443	0.193
0.28	0.310	0.308	0.744	0.376	0.316	0.260	0.400	0.248
0.29	0.367	0.300	0.888	0.170	0.470	0.194	0.579	0.162
0.3	0.386	0.334	0.842	0.197	0.435	0.289	0.514	0.230
0.31	0.319	0.304	0.848	0.238	0.402	0.240	0.496	0.226
0.32	0.390	0.299	0.844	0.271	0.375	0.220	0.464	0.203
0.33	0.365	0.315	0.859	0.245	0.420	0.254	0.518	0.234
0.34	0.311	0.288	0.718	0.332	0.346	0.236	0.425	0.241
0.35	0.456	0.361	0.784	0.279	0.458	0.331	0.496	0.254
0.36	0.365	0.292	0.743	0.316	0.359	0.234	0.445	0.235
0.37	0.583	0.305	0.822	0.280	0.455	0.304	0.527	0.240
0.38	0.551	0.309	0.669	0.371	0.423	0.315	0.466	0.274
0.39	0.516	0.351	0.752	0.345	0.469	0.299	0.535	0.271
0.4	0.473	0.352	0.775	0.333	0.423	0.268	0.511	0.266
0.41	0.604	0.266	0.718	0.329	0.485	0.277	0.555	0.263
0.42	0.621	0.318	0.667	0.306	0.530	0.340	0.521	0.264
0.43	0.569	0.302	0.515	0.410	0.361	0.332	0.386	0.308
0.44	0.538	0.307	0.571	0.273	0.467	0.301	0.451	0.216
0.45	0.599	0.317	0.493	0.313	0.434	0.337	0.410	0.259
0.46	0.647	0.284	0.465	0.362	0.382	0.331	0.381	0.283
0.47	0.663	0.217	0.586	0.315	0.441	0.268	0.470	0.227
0.48	0.704	0.193	0.394	0.334	0.391	0.357	0.358	0.290
0.49	0.754	0.241	0.353	0.334	0.332	0.292	0.309	0.251
0.5	0.658	0.206	0.491	0.284	0.444	0.236	0.444	0.223
0.51	0.752	0.241	0.299	0.266	0.393	0.342	0.321	0.265
0.52	0.778	0.238	0.297	0.264	0.395	0.297	0.310	0.219
0.53	0.799	0.164	0.276	0.304	0.329	0.347	0.268	0.258
0.54	0.806	0.192	0.268	0.255	0.364	0.307	0.281	0.225
0.55	0.811	0.188	0.286	0.259	0.388	0.339	0.299	0.238
0.56	0.852	0.129	0.277	0.288	0.344	0.304	0.279	0.238
0.57	0.834	0.171	0.292	0.259	0.358	0.279	0.308	0.239
0.58	0.883	0.142	0.264	0.301	0.412	0.389	0.276	0.259
0.59	0.832	0.150	0.368	0.264	0.535	0.313	0.405	0.236

Continued on next page

**Threshold**	**Specificity**		**Sensitivity**		**Precision**		**F1 Score**	
	Mean	SD	Mean	SD	Mean	SD	Mean	SD
0.6	0.790	0.268	0.188	0.180	0.380	0.355	0.223	0.206
0.61	0.942	0.091	0.114	0.210	0.297	0.377	0.134	0.181
0.62	0.898	0.168	0.089	0.206	0.201	0.340	0.109	0.216
0.63	0.883	0.184	0.102	0.245	0.126	0.258	0.100	0.213
0.64	0.881	0.205	0.098	0.229	0.136	0.268	0.104	0.215
0.65	0.939	0.111	0.033	0.114	0.046	0.144	0.038	0.125
0.66	0.939	0.122	0.061	0.146	0.082	0.174	0.068	0.155
0.67	0.955	0.108	0.076	0.177	0.131	0.308	0.094	0.216
0.68	0.983	0.049	0.019	0.094	0.019	0.094	0.019	0.094
0.69	0.959	0.076	0.036	0.123	0.047	0.160	0.040	0.136
0.7	0.988	0.042	0.019	0.096	0.019	0.096	0.019	0.096
0.71	0.993	0.024	0.019	0.094	0.025	0.126	0.021	0.108
0.72	0.988	0.036	0.055	0.141	0.107	0.268	0.069	0.173
0.73	0.996	0.021	0.015	0.077	0.026	0.128	0.019	0.096
0.74	0.992	0.029	0.036	0.123	0.053	0.181	0.043	0.146
0.75	0.986	0.036	0.037	0.116	0.061	0.185	0.046	0.140
0.76	0.990	0.030	0.048	0.140	0.065	0.191	0.055	0.161
0.77	0.988	0.036	0.055	0.135	0.107	0.268	0.072	0.177
0.78	0.996	0.022	0.008	0.039	0.020	0.098	0.011	0.056
0.79	0.993	0.025	0.007	0.036	0.017	0.091	0.010	0.052
0.8	1.000	0.000	0.045	0.102	0.167	0.373	0.071	0.159
0.81	1.000	0.000	0.022	0.061	0.115	0.319	0.037	0.102
0.82	1.000	0.000	0.016	0.058	0.071	0.258	0.026	0.095
0.83	1.000	0.000	0.006	0.030	0.034	0.182	0.010	0.052
0.84	1.000	0.000	0.024	0.069	0.111	0.314	0.040	0.112
0.85	1.000	0.000	0.008	0.035	0.048	0.213	0.014	0.061
0.86	1.000	0.000	0.017	0.059	0.083	0.276	0.029	0.096
0.87	1.000	0.000	0.017	0.059	0.074	0.262	0.027	0.096
0.88	1.000	0.000	0.008	0.038	0.038	0.192	0.013	0.064
0.89	1.000	0.000	0.000	0.000	0.000	0.000	0.000	0.000
0.9	1.000	0.000	0.000	0.000	0.000	0.000	0.000	0.000

Table S16: Evaluation metrics by frequency and threshold of CNN SSL model for ID 27. The AUC score is 0.613.

**Threshold**	**Specificity**		**Sensitivity**		**Precision**		**F1 Score**	
	Mean	SD	Mean	SD	Mean	SD	Mean	SD
0.1	0.000	0.000	1.000	0.000	0.305	0.186	0.438	0.207
0.11	0.000	0.000	1.000	0.000	0.293	0.153	0.433	0.180
0.12	0.000	0.000	1.000	0.000	0.378	0.182	0.524	0.189
0.13	0.000	0.000	1.000	0.000	0.277	0.162	0.410	0.194
0.14	0.000	0.000	1.000	0.000	0.300	0.195	0.431	0.212
0.15	0.000	0.000	1.000	0.000	0.330	0.191	0.465	0.220
0.16	0.000	0.000	1.000	0.000	0.327	0.176	0.468	0.184
0.17	0.000	0.000	1.000	0.000	0.360	0.206	0.498	0.210
0.18	0.000	0.000	1.000	0.000	0.293	0.155	0.431	0.185
0.19	0.000	0.000	1.000	0.000	0.304	0.172	0.440	0.198
0.2	0.000	0.000	1.000	0.000	0.317	0.161	0.460	0.185
0.21	0.000	0.000	1.000	0.000	0.297	0.110	0.447	0.128
0.22	0.000	0.000	1.000	0.000	0.302	0.174	0.439	0.182
0.23	0.000	0.000	1.000	0.000	0.311	0.175	0.448	0.204
0.24	0.000	0.000	1.000	0.000	0.307	0.177	0.444	0.189
0.25	0.000	0.000	1.000	0.000	0.313	0.212	0.440	0.230
0.26	0.000	0.000	1.000	0.000	0.389	0.174	0.538	0.175
0.27	0.000	0.000	1.000	0.000	0.302	0.199	0.430	0.229
0.28	0.000	0.000	0.973	0.074	0.400	0.217	0.536	0.211
0.29	0.000	0.000	0.967	0.064	0.311	0.204	0.433	0.221
0.3	0.000	0.000	0.982	0.055	0.309	0.192	0.438	0.208
0.31	0.000	0.000	0.947	0.100	0.289	0.210	0.404	0.233
0.32	0.022	0.046	0.947	0.129	0.296	0.159	0.428	0.183
0.33	0.055	0.063	0.879	0.229	0.290	0.170	0.409	0.185
0.34	0.044	0.064	0.851	0.264	0.254	0.165	0.364	0.197
0.35	0.058	0.094	0.875	0.253	0.272	0.168	0.391	0.200
0.36	0.076	0.142	0.899	0.189	0.297	0.163	0.417	0.185
0.37	0.103	0.171	0.901	0.183	0.304	0.217	0.414	0.230
0.38	0.134	0.183	0.900	0.159	0.314	0.183	0.435	0.189
0.39	0.201	0.205	0.888	0.244	0.395	0.256	0.506	0.267
0.4	0.243	0.257	0.877	0.231	0.415	0.282	0.515	0.276
0.41	0.355	0.259	0.873	0.236	0.384	0.215	0.497	0.214
0.42	0.314	0.260	0.832	0.275	0.357	0.250	0.457	0.253
0.43	0.348	0.293	0.875	0.238	0.406	0.269	0.506	0.267
0.44	0.486	0.301	0.785	0.254	0.459	0.262	0.526	0.228
0.45	0.515	0.266	0.792	0.291	0.491	0.196	0.563	0.186
0.46	0.561	0.251	0.735	0.302	0.413	0.237	0.494	0.228
0.47	0.660	0.268	0.582	0.361	0.399	0.351	0.362	0.234
0.48	0.715	0.254	0.507	0.341	0.494	0.339	0.398	0.191
0.49	0.688	0.276	0.522	0.354	0.377	0.248	0.371	0.208
0.5	0.758	0.212	0.349	0.378	0.420	0.372	0.311	0.275
0.51	0.836	0.171	0.240	0.315	0.240	0.329	0.203	0.249
0.52	0.827	0.210	0.256	0.233	0.324	0.314	0.271	0.241
0.53	0.873	0.138	0.191	0.225	0.228	0.273	0.195	0.219
0.54	0.946	0.088	0.091	0.150	0.179	0.306	0.109	0.174
0.55	0.888	0.112	0.089	0.158	0.149	0.257	0.102	0.172
0.56	0.954	0.091	0.098	0.146	0.321	0.427	0.135	0.186
0.57	0.945	0.088	0.054	0.111	0.167	0.333	0.071	0.136
0.58	0.948	0.109	0.053	0.096	0.226	0.386	0.077	0.128
0.59	0.951	0.085	0.104	0.163	0.212	0.335	0.134	0.205
0.6	0.972	0.067	0.052	0.101	0.167	0.320	0.076	0.143
0.61	0.987	0.046	0.069	0.140	0.231	0.398	0.097	0.178

Continued on next page

Threshold	Specificity		Sensitivity		Precision		F1 Score	
	Mean	SD	Mean	SD	Mean	SD	Mean	SD
0.62	0.958	0.073	0.053	0.135	0.113	0.256	0.063	0.147
0.63	0.992	0.027	0.056	0.125	0.190	0.372	0.078	0.162
0.64	0.979	0.043	0.056	0.127	0.147	0.315	0.075	0.164
0.65	0.969	0.054	0.095	0.162	0.208	0.331	0.124	0.204
0.66	0.978	0.049	0.056	0.121	0.113	0.244	0.075	0.160
0.67	0.997	0.017	0.012	0.063	0.019	0.094	0.015	0.076
0.68	0.993	0.027	0.031	0.115	0.046	0.169	0.037	0.136
0.69	0.996	0.019	0.019	0.094	0.025	0.126	0.021	0.108
0.7	0.996	0.022	0.048	0.130	0.107	0.294	0.066	0.178
0.71	0.996	0.021	0.015	0.076	0.025	0.126	0.019	0.094
0.72	0.989	0.031	0.033	0.119	0.049	0.175	0.040	0.141
0.73	1.000	0.000	0.046	0.128	0.115	0.319	0.066	0.183
0.74	1.000	0.000	0.010	0.048	0.038	0.192	0.015	0.077
0.75	1.000	0.000	0.049	0.142	0.111	0.314	0.068	0.194
0.76	1.000	0.000	0.008	0.038	0.038	0.192	0.013	0.064
0.77	1.000	0.000	0.029	0.072	0.143	0.350	0.048	0.119
0.78	1.000	0.000	0.000	0.000	0.000	0.000	0.000	0.000
0.79	1.000	0.000	0.000	0.000	0.000	0.000	0.000	0.000
0.8	1.000	0.000	0.000	0.000	0.000	0.000	0.000	0.000
0.81	1.000	0.000	0.000	0.000	0.000	0.000	0.000	0.000
0.82	1.000	0.000	0.000	0.000	0.000	0.000	0.000	0.000
0.83	1.000	0.000	0.000	0.000	0.000	0.000	0.000	0.000
0.84	1.000	0.000	0.000	0.000	0.000	0.000	0.000	0.000
0.85	1.000	0.000	0.000	0.000	0.000	0.000	0.000	0.000
0.86	1.000	0.000	0.000	0.000	0.000	0.000	0.000	0.000
0.87	1.000	0.000	0.000	0.000	0.000	0.000	0.000	0.000
0.88	1.000	0.000	0.000	0.000	0.000	0.000	0.000	0.000
0.89	1.000	0.000	0.000	0.000	0.000	0.000	0.000	0.000
0.9	1.000	0.000	0.000	0.000	0.000	0.000	0.000	0.000
